# Effects of fundamental social motives on environmental variation of compliance during pandemics

**DOI:** 10.1017/ehs.2026.10062

**Published:** 2026-07-03

**Authors:** Ruoting Liu, Tingshao Zhu, Xuefeng Chen, Yan-Mei Li

**Affiliations:** Institute of Psychology Chinese Academy of Sciences, Beijing, China

**Keywords:** fundamental social motives, familial bonds, infectious disease, socioeconomic development, compliance

## Abstract

Compliance with epidemic prevention norms has been found to be higher in developing regions than in developed regions; however, the nature and underlying mechanisms remain unclear. We propose that socioeconomic development of environments changes the adaptive benefits of fundamental social motives, especially disease avoidance and familial motives, which shape variations in compliance during pandemics. To examine the effects of these motives on the relationship between socioeconomic environments and compliance, we conducted three studies measuring environmental socioeconomic development with the Human Development Index (HDI). Study 1 (with two datasets: *N* = 43,244, 53 countries; *N* = 94,657, 71 countries) revealed a stable negative correlation between country-level HDI and compliance, even after controlling disease severity, government responses, and individualism. Studies 2 (an analysis of social media text data; *N* = 22,588, 31 provinces) and 3 (a large-sample survey; *N* = 6,122, 31 cities) replicated this correlation in China at the provincial and city levels, and identified disease avoidance and familial motives as mediators. These findings provide evidence for how socioeconomic environments shape compliance during pandemics, highlight the importance of familial motives alongside disease avoidance, and offer insights into tailoring public health strategies across diverse environments.

## Graphical abstract

**Figure:**
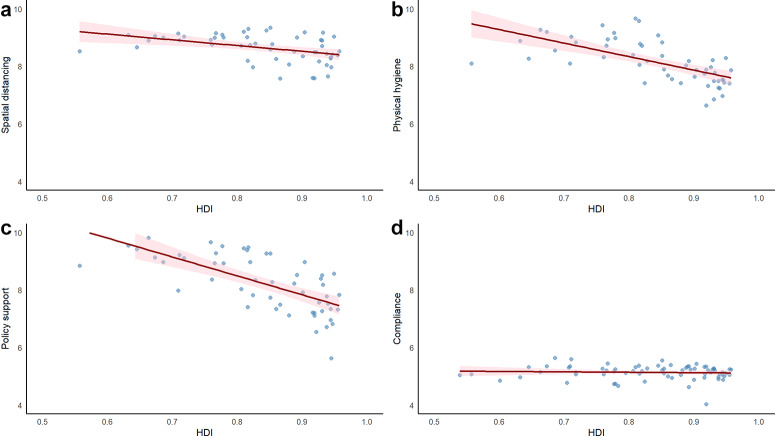

## Social media summary

Three studies showed that higher socioeconomic development at national, provincial, and city levels predicts lower compliance with pandemic norms, driven by reduced disease avoidance and familial motives.

## 1. Introduction

Infectious diseases have consistently posed a substantial threat to human survival and reproduction throughout evolutionary history (Inhorn & Brown, 1990; Wolfe et al., 2007). Norms are shared rules and expectations that guide behaviour in a society or group, maintaining social order and shaping interactions (Cialdini et al., 1990). Epidemic prevention norms, typically conceptualized as prescriptive (or injunctive) norms, refer to societal standards regarding what individuals ought to do and function to help people cope with the threat of infectious diseases (Ellickson, 1999; Fabrega, 1997; Murray et al., 2011). Compliance with prevention norms is crucial for containing disease spread and mitigating economic and political disruptions, particularly in the absence of effective medical treatments or vaccines (Bish & Michie, 2010; Hatchett et al., 2007; Jin et al., 2020). Recent studies have found that people in developing countries are more compliant with epidemic prevention norms than those in developed countries (Aamir et al., 2022; Lin et al., 2021). These findings suggest a potential link between levels of socioeconomic development and individual compliance, yet few studies have systematically examined this relationship.

An evolutionary perspective offers a rich explanation for the association between environment and human behaviours, considering behaviours as adaptations to the environment (Ellis et al., 2009; Griskevicius et al., 2011). Disparities in environmental characteristics have led to the adoption of distinct adaptive psychological strategies (Brandstätter et al., 2016; Eom et al., 2019; Pedersen et al., 2022). The fundamental social motives summarize a series of psychological strategies that have evolved in response to recurrent challenges and opportunities, initiating and organizing behaviours in social life (Kenrick et al., 2010; Neel et al., 2016). Empirical evidence has shown these motives are closely linked to compliance during pandemics (Liu et al., 2023; Pick et al., 2022). The present study aims to systematically examine the relationship between socioeconomic development of environments and individual compliance with epidemic prevention norms while exploring the underlying psychological mechanisms through the lens of fundamental social motives.

### 1.1. Variations in compliance with epidemic prevention norms across socioeconomic development environments

From the perspective of socioecological and evolutionary psychology, socioeconomic development reflects levels of resource availability and mortality risk, which are fundamental environmental factors and important ecological cues throughout evolutionary history. These cues set up selection pressures that shape adaptive strategies and behaviours (Griskevicius et al., 2013; Gurven, 2018; Jonason et al., 2016). Regions with lower levels of socioeconomic development face greater challenges in epidemic control, often due to shortages of social and economic resources (such as medical services and health insurance), resulting in a higher mortality risk (Galvan et al., 2020). In contrast, regions with higher socioeconomic development tend to have more abundant healthcare resources and lower numbers of pandemic-related cases and deaths (Galvan et al., 2020). This reduced mortality risk may lessen the urgency and importance of strict norm compliance aimed at avoiding infection and death from the pandemic.

In line with this notion, multiple studies have shown that people in lower socioeconomic environments demonstrate greater compliance with prevention norms, such as maintaining social distancing and reducing outdoor activities during pandemics. Lin et al. (2021), using survey data from 66 countries/territories, found that people in countries with lower Human Development Index (HDI) exhibited higher compliance. Aamir et al. (2022) observed that, compared to developed countries, people from developing countries were more compliant with all COVID-19 prevention and control measures except handwashing. However, it remains unclear whether the effects of socioeconomic development are robust across different measures, across regions beyond the country level, and after controlling for other ecological variables, and the underlying psychological process has yet to be investigated.

A large body of literature presumes that natural selection shapes human social motives in response to adaptive challenges in group living (Buss, 1991; Gigerenzer, 2000; Haselton & Nettle, 2006; Kenrick et al., 2010; Neel et al., 2016; Neuberg et al., 2010). Variations in compliance across environments with different levels of socioeconomic development during pandemics may result from the increased importance of certain social motives in mitigating threats in specific ecological contexts.

### 1.2. Effects of socioeconomic environments on disease avoidance and familial fundamental social motives during pandemics

Fundamental social motives conceptually identify human psychological strategies inherited for coping with recurrent challenges of group living and encompass seven motive systems: self-protection, disease avoidance, affiliation, status seeking, mate seeking, mate retention, and kin care (Neel et al., 2016; Schaller et al., 2017). Cost-benefit analyses of each motive, shaped by environmental characteristics, determine motivational priorities (Jonason et al., 2016; Kenrick et al., 2010).

#### Disease avoidance and familial motives

*Disease avoidance* has been found to guide behaviours that minimize direct or indirect contact with infectious individuals during pandemics, thereby reducing infection risk (Gul et al., 2022; Tybur et al., 2020). Studies about epidemics have shown that stronger disease avoidance motives predict greater compliance with prevention norms (DiGiovanni et al., 2004; Sawada et al., 2018; Shook et al., 2020).

Mate retention (i.e., maintaining a long-term romantic/sexual partnership), kin care (family), and kin care (child) motives are collectively referred to as *familial motives* because they are closely related to the formation and maintenance of long-term familial bonds that promote reproductive success (Ko et al., 2019; Martin et al., 2020; Sear & Coall, 2011; West & Gardner, 2013). Altruism research has indicated that individuals are more likely to aid offspring, blood relatives, and romantic partners, especially those with biological children, in life-or-death situations (Burnstein et al., 1994; Hernández Blasi, 2023; Poirotte & Charpentier, 2020). The implication is that familial motives are critical psychological strategies that increase opportunities for reproductive success during crises. Studies on COVID-19 provide indirect evidence supporting this interpretation. During the pandemic, familial motives remained a strong priority (Pick et al., 2022) and long-term familial bonds reduced psychological distress (Randall et al., 2022).

Pandemics pose serious threats to human survival and reproduction (Pyszczynski et al., 2021). Under such conditions, face-to-face interactions and social engagement with others, particularly unfamiliar individuals, can increase infection risk and threaten survival (Jarvis et al., 2020; Murray & Schaller, 2016). As a result, individuals may deprioritize goals such as wealth and status pursuit, group belonging, and mate seeking, particularly in the initial phase when targeted antivirals or vaccines are unavailable (Ackerman et al., 2018; Brown & Sacco, 2016; Gruijters et al., 2016; Mortensen et al., 2010; Young et al., 2021).

Taken together, the present study focuses on disease avoidance and familial motives, which may provide greater adaptive benefits during pandemics and serve as significant drivers of compliance with epidemic prevention norms.

#### Effects of socioeconomic development

The adaptive benefits of disease avoidance and familial motives may vary with socioeconomic development levels across environments in the context of prevalent infectious diseases. Studies conducted during the COVID-19 pandemic have revealed that infectious diseases exacerbate mortality risk more severely in areas with low socioeconomic development than in developed areas, primarily due to resource constraints (Ameye et al., 2022; Ji et al., 2020; Mirahmadizadeh et al., 2022). These findings imply that during pandemics, the threats of resource scarcity and mortality become more salient in environments with lower socioeconomic development, where it is difficult for people to obtain support from social security systems or help from non-kin others, and where treatment costs for infection are high. In such environments, disease avoidance and interdependence with family members offer more benefits for survival and reproduction. Cross-country and individual difference research provides indirect evidence for environmental influences on disease avoidance. Karakulak et al. (2023) observed that individuals in low socioeconomic areas reported higher levels of perceived vulnerability to disease (PVD) during the pandemic. PVD is closely linked to the disease avoidance motive (Díaz et al., 2016; Schaller, 2014). The environmental effects may also manifest in familial motives. People in less socioeconomically developed regions tend to rely more on long-term familial bonds to maximize resources for raising offspring and enhancing reproductive fitness (Kaplan et al., 2000; Sear & Mace, 2008). Research on cultural evolution has shown that kinship bonds were stronger in preindustrial times, which were characterized by limited socioeconomic development, than in the contemporary period (Bahrami-Rad et al., 2022; Enke, 2019). These studies suggest that people allocate more energy to familial goals to adapt to environments with lower socioeconomic development. Therefore, during a pandemic, the lower the level of socioeconomic development in an environment, the stronger the fundamental motives for disease avoidance and familial bond maintenance.

### 1.3. Social motives and compliance with epidemic prevention norms

Disease avoidance and familial motives may be essential for understanding variations in compliance with prevention norms during pandemics. These motives initiate and manage the compliance behaviours that support underlying goals and enhance fitness in an epidemic. Empirical evidence reveals that disease avoidance improves compliance. Activation of disease avoidance goals has been identified as a predictor of compliance with epidemic prevention norms (Sacco et al., 2014). COVID-19 research has shown that the disease avoidance motive leads individuals to actively adopt social distancing and hygiene behaviours, even after controlling for variables such as pathogen disgust sensitivity, personality, perceived risk, and pandemic knowledge (Gul et al., 2022; Makhanova et al., 2021). In addition to disease avoidance, familial motives may also have similar effects on compliance. Studies based on American or Australian samples have shown that during epidemics (e.g., COVID-19 or pandemic influenza), concern for family members’ health and survival is positively correlated with willingness to accept vaccination, self-isolation, and mask-wearing (Cohen et al., 2020; Taylor et al., 2009). These findings suggest that familial motives are important psychological strategies to initiate compliance with epidemic prevention norms.

### 1.4. The mediating role of fundamental social motives on the relationship between socioeconomic development and compliance

Building on what has been discussed above, variations in compliance with prevention norms across environments with differing levels of socioeconomic development during pandemics may derive from disease avoidance and familial motives. These may reflect how ancestors solved motivational trade-offs based on adaptive benefits and costs in various natural and social ecologies. During epidemics, ancestors living in environments characterized by limited medical resources and high mortality – conditions mirrored in contemporary regions with lower levels of socioeconomic development – would have experienced more adaptive benefits from disease avoidance and maintaining long-term familial bonds. Compliance with prevention norms varies across environments with different levels of socioeconomic development, serving as behavioural expressions of these underlying motivational systems. Therefore, we expect that the relationship between socioeconomic development and compliance with prevention norms will be mediated by disease avoidance and familial fundamental social motives.

### 1.5. Current research

**Figure 1.**
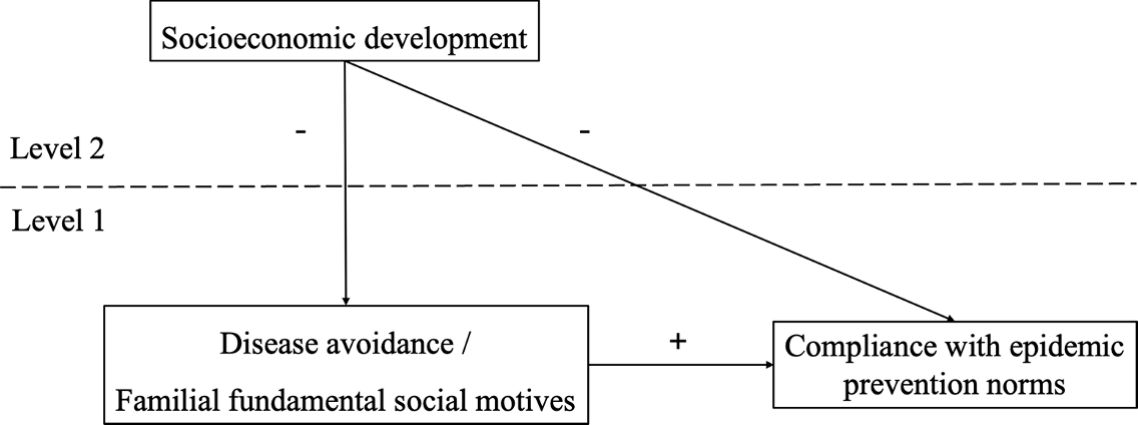
Hypothesized model.

In summary, the current research test (1) whether regional socioeconomic development stably predicts individuals’ compliance with epidemic prevention norms and (2) whether disease avoidance and familial motives mediate this relationship (see Figure 1). In Study 1, we used secondary data to examine the relationship between national socioeconomic development and individual compliance. Studies 2 (social media text data analysis) and 3 (large-sample survey) investigated the mediating effects of disease avoidance and familial motives on this relationship at the provincial and city levels, respectively.

## 2. Study 1

We used the HDI to measure regional socioeconomic development. Study 1 investigated the relationship between the HDI and compliance with epidemic prevention norms using two publicly available cross-national datasets collected during the early phase of the COVID-19 pandemic. The first dataset was drawn from a survey conducted by Van Bavel et al. (2022) (Data A), and the second was sourced from the COVIDiSTRESS Global Survey (Data B).

### 2.1. Methods

#### Participants

We excluded data from countries with missing country-level indicators or sample sizes below five (Bentler & Chou, 1987). The present analysis included Data A, which comprised 43,244 participants from 53 countries/territories (*M*_age_ = 43.76, *SD*_age_ = 16.20; range: 18–100 years; 52.1% female), and Data B, which comprised 94,657 participants from 71 countries/territories (*M*_age_ = 39.89, *SD*_age_ = 14.12; range: 18–110 years; 72.5% female).

#### Materials

***Socioeconomic development***: The HDI from the *Human Development Report 2020* (United Nations Development Programme, 2020) was used to measure regional socioeconomic development. It is a composite index that integrates three sub-dimensions: life expectancy at birth (Health), mean years of schooling (Education), and gross national income per capita (Income), with broad coverage and regular annual updates (Stanton, 2007).

***Compliance with epidemic prevention norms***: Data A measured self-reported compliance with prevention norms using three indicators: spatial distancing (four items, e.g., ‘Staying at home as much as practically possible’), physical hygiene (five items, e.g., ‘Washing my hands longer than usual’), and policy support (five items, e.g., ‘In favor of closing all schools and universities’). Responses were rated on an 11-point slider scale ranging from 0 (strongly disagree) to 10 (strongly agree). We computed the average scores for the items within each indicator separately. Data B used two items (e.g., ‘I have done everything I could possibly do as an individual to reduce the spread of the coronavirus’), each rated on a 6-point Likert scale (1 = strongly disagree, 6 = strongly agree). The average score across the two items was used to indicate overall compliance.

***Control variables***: Prior studies have shown that the individualism–collectivism dimension (Courtney et al., 2022), the implementation of non-pharmaceutical interventions (Pak et al., 2021), the severity of the outbreak (Murray et al., 2011), and trust in institutions (Bargain & Aminjonov, 2020) influence individuals’ compliance during pandemics. In addition, population density and demographic variables such as sex and age play a crucial role in shaping threat perception and behavioural responses (Arbel et al., 2022; Bish & Michie, 2010). We therefore included sex, age, and trust in institutions (in Data B only) as individual-level covariates, along with COVID-19 severity (measured by confirmed cases per million and case fatality rate [CFR]), government response, individualism, and population density as country-level covariates. The details of the measures and sources of these covariates are available in Table S1 in the Supplemental Materials. To minimize the effect of causal inversion, data on COVID-19 severity and government response were obtained from the first day of the two surveys (Data A: April 1, 2020; Data B: March 26, 2020).

#### Statistical analyses

All statistical analyses were performed using *R* (R Core Team, 2024; Version 4.2). Pearson’s correlations across variables were computed. Given the nested data structure, hierarchical linear models (HLMs) were constructed using the *lmer* function in the *lmerTest* package (Kuznetsova et al., 2017). All variables were standardized in the HLMs. Either grand- or group-mean centring can be used to examine the differential influence of a variable at Levels 1 and 2 (Enders & Tofighi, 2007). Science grand-mean centring offers a more parsimonious interpretation of the intercept, and all continuous variables were grand-mean-centred. Because of the distinct measurements in Data A and Data B, they were analysed separately. Our data are available on the OSF (https://osf.io/cxha6/?view_only=85d67626c08d414ca73ba87764ef62c2).

### 2.2. Results

**Figure 2.**
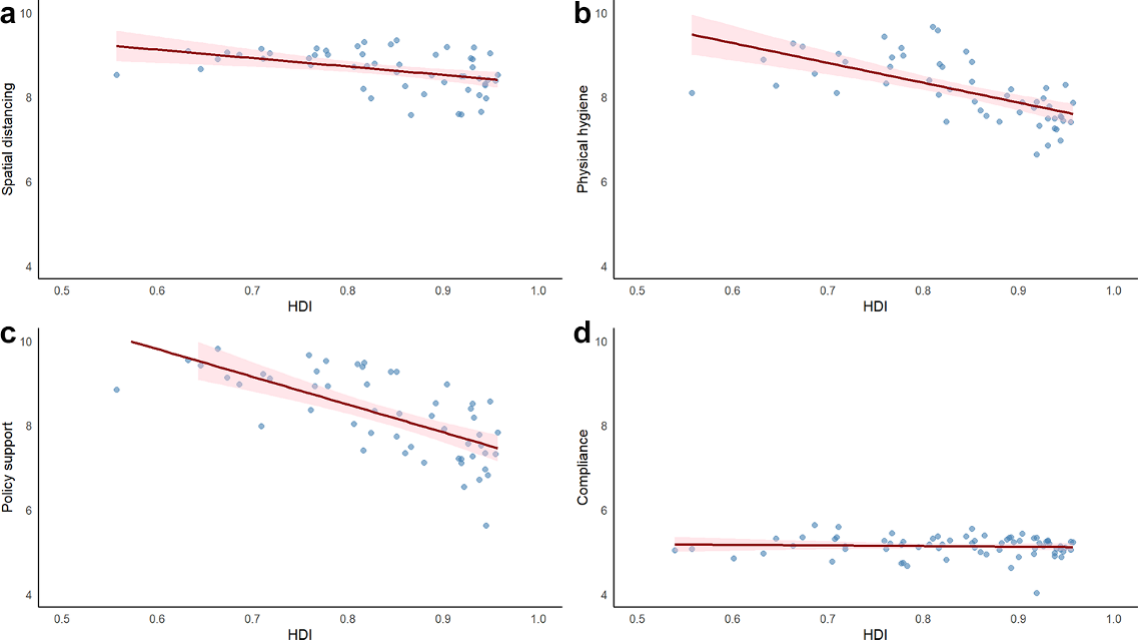
The correlation between country-level HDI and averages for compliance with epidemic prevention norms. The results in sub-figures (a), (b), and (c) are from Data A, while sub-figure (d) presents data from Data B. Figure 2 long description.

As predicted, country-level HDI was correlated negatively with spatial distancing (*r* = −0.42, *p* = 0.002), physical hygiene (*r* = −0.64, *p* < 0.001), and policy support (*r* = −0.67, *p* < 0.001) in Data A. The HDI was not significantly correlated with the comprehensive compliance index (*r* = −0.06, *p* = 0.613) in Data B (Figure 2). Descriptive statistics and the correlation matrix of all variables are presented in Table S2 and Figure S1, respectively, and the geographic distribution of compliance across countries is shown in Figure S2.

The intraclass correlation (ICC) values in this study were 0.069 for comprehensive compliance in Data B, 0.070 for spatial distancing in Data A, 0.133 for physical hygiene in Data A, and 0.177 for policy support in Data A. HLMs were used because the ICCs indicated non-negligible between-group variance, suggesting meaningful variation across countries.

The results of the HLMs are presented in Table 1. Model 1 included only the control variables. Based on this, Model 2 added the HDI to the intercept term. As expected, the HDI was significantly negatively associated with spatial distancing (*β* = −0.130, *p* = 0.029), policy support (*β* = −0.169, *p* = 0.035), and the comprehensive compliance index (*β* = −0.093, *p* = 0.011), and was marginally negatively associated with physical hygiene (*β* = −0.116, *p* = 0.069). In addition, consistent with previous research findings, older individuals, females, and those with greater trust in institutions tended to show a stronger inclination to comply with epidemic prevention norms, whereas individuals living in regions with a higher CFR, stricter governmental responses, and lower levels of individualism also showed higher compliance levels (Chen et al., 2021; Kim & Kwan, 2021; Margraf et al., 2020). Notably, the effects of COVID-19 severity, government response, and individualism on compliance appeared to diminish or even disappear when the HDI was factored into the model, and the association between the HDI and compliance was more pronounced and statistically significant than that between other factors. To examine potential confounding by geographic and sociocultural clustering, we conducted Moran’s *I* tests and re-estimated models controlling for capital latitude and longitude as robustness checks. The results show that HDI remained a significant and negative predictor of compliance, indicating the association is not attributable to spatial dependence (see Section 1.3 in the Supplementary Materials for details).

**Table 1.** Hierarchical linear model analysis in Study 1 Table 1 long description.

	Data A (*N* = 43,244, *n* = 53)						Data B (*N* = 94,657, *n* = 71)	
	Spatial distancing		Physical hygiene		Policy support		Compliance	
	Model 1	Model 2	Model 1	Model 2	Model 1	Model 2	Model 1	Model 2
Variables	*β* (*SE*)	*β* (*SE*)	*β* (*SE*)	*β* (*SE*)	*β* (*SE*)	*β* (*SE*)	*β* (*SE*)	*β* (*SE*)
Constant	8.572***	8.567***	7.962***	7.957***	8.007***	7.999***	5.139***	5.112***
	(0.062)	(0.059)	(0.071)	(0.069)	(0.108)	(0.104)	(0.032)	(0.032)
**Level 1**								
Age	0.114***	0.114***	0.070***	0.070***	0.037***	0.037***	0.142***	0.142***
	(0.005)	(0.005)	(0.005)	(0.005)	(0.005)	(0.005)	(0.003)	(0.003)
Gender	0.137***	0.137***	0.146***	0.146***	0.090***	0.090***	0.111***	0.111***
	(0.005)	(0.005)	(0.005)	(0.005)	(0.004)	(0.004)	(0.003)	(0.003)
Institutional trust							0.070***	0.070***
							(0.004)	(0.004)
**Level 2**								
Confirmed cases/million	−0.043	0.055	−0.064	0.023	−0.141*	−0.014	0.004	0.083✝
	(0.042)	(0.059)	(0.044)	(0.064)	(0.056)	(0.080)	(0.034)	(0.044)
CFR	0.075*	0.051	0.050	0.028	0.086✝	0.054	0.006	−0.011
	(0.034)	(0.034)	(0.036)	(0.037)	(0.045)	(0.046)	(0.025)	(0.025)
Government response	0.079*	0.061✝	0.046	0.031	0.072	0.049	0.113**	0.096**
	(0.035)	(0.035)	(0.037)	(0.037)	(0.047	(0.047)	(0.037)	(0.035)
Individualism	−0.070✝	−0.028	−0.212***	−0.174***	−0.161**	−0.107✝	−0.058✝	−0.022
	(0.039)	(0.042)	(0.042)	(0.045)	(0.052)	(0.056)	(0.030)	(0.032)
Population density	0.008	0.018	−0.067*	−0.058✝	−0.013	−0.000	−0.004	−0.002
	(0.030)	(0.029)	(0.031)	(0.031)	(0.040)	(0.039)	(0.007)	(0.07)
HDI		−0.130*		−0.116✝		−0.169*		−0.093*
		(0.058)		(0.062)		(0.078)		(0.036)
*R*^2^_marginal_	0.042	0.046	0.082	0.083	0.076	0.079	0.048	0.051
*R*^2^_conditional_	0.098	0.097	0.145	0.143	0.180	0.176	0.111	0.107
*χ*^2^	–	5.55*	–	3.87*	–	5.20*	–	7.13**

*Notes*: Gender: 1 = female, 0 = male/other/no reply. *β* = standardized coefficient; *R*^2^_marginal_ = variance explained by fixed factors; *R*^2^_conditional_ = variance explained by both fixed and random factors. ✝*p* < 0.1, *p < 0.05, **p < 0.01, ***p < 0.001. All continuous variables were grand-mean-centred.

In addition, as an exploratory analysis, we replaced the overall HDI with its three sub-dimensions in the HLMs to examine which component most strongly predicts compliance with epidemic prevention norms during pandemics (see Table S15). However, the three sub-dimensions were highly correlated with each other (*r*s > 0.62), and the multicollinearity diagnostics indicated a high variance inflation factor for the income sub-dimension (VIFs > 11.79 in Data A; VIF = 6.63 in Data B). Therefore, to avoid multicollinearity and potential interpretational ambiguity, we retained the composite HDI index as the primary predictor in subsequent analyses.

### 2.3. Discussion

The findings of Study 1 revealed a consistent and significant negative correlation between country-level HDI and compliance: the higher a country’s HDI, the lower its level of compliance with epidemic prevention norms. This relationship was found to be stronger than those between other variables and compliance across datasets and measures, suggesting an important role of socioeconomic development in predicting compliance during pandemics. Therefore, it is worthwhile to further explore the psychological mechanisms underlying this connection, and we thus conducted Study 2.

## 3. Study 2

In Study 2, data from Sina Weibo (a popular social media platform in China) were used to examine the relationship between province-level HDI and individual compliance with prevention norms as well as the mediating roles of disease avoidance and familial motives.

### 3.1. Methods

#### Lexicons

***Compliance***: We constructed a lexicon of compliance with epidemic prevention norms based on several official public health guidelines issued for pandemic protection (see Table S5 for details). As compliance-related keywords may also appear in expressions of noncompliance (e.g., ‘wearing a mask’ in the phrase ‘not wearing a mask’), we compiled a set of words and phrases related to noncompliance to ensure the accuracy of capturing compliance tendencies. After expanding the lexicon based on common Weibo expressions, eliminating repetitive words, and filtering and evaluating by 3 experts, we identified 380 keywords related to noncompliance (e.g., ‘no need to wash your hands’, ‘vaccines don’t work’, and ‘hate to stay at home’). The complete list of noncompliance keywords is available on the OSF (https://osf.io/cxha6/?view_only=85d67626c08d414ca73ba87764ef62c2).

***Motives***: We constructed lexicons of motives based on validated psychological scales and prior lexicon-based research (see Table S5 for details). Because of the significant overlap in the keywords for kin care (family) and kin care (child) (e.g., parents, son, daughter), we integrated the keywords of these 2 motives, resulting in 3 lexicons: disease avoidance (387 words), mate retention (317 words), and kin care (310 words).

#### Weibo data

Data were obtained from the Weibo User Pool of the Computational Cyber Psychology Lab. To reduce the impact of strict prevention measures on compliance during the early stages of the epidemic, we defined the observation period from March 25, 2020 (when Hubei Province lifted its lockdown) to June 25, 2020. We collected Weibo posts containing noncompliance keywords from a data pool posted during this period. We then filtered the samples and extracted valid users according to the following criteria: (1) nonpublic and noncommercial accounts, (2) published text data of more than 3 kb, and (3) regional authentication in mainland China. The final sample consisted of 22,588 users from 31 provinces (*M*_sample_ = 728.68; *SD*_sample_ = 973.60).

#### Word frequency analysis

We used word frequency analysis to quantify Weibo user compliance. *Jieba* Chinese text segmentation (a *Python* module) was used to segment and extract text data. By importing the noncompliance lexicon, we calculated the frequency of keywords, reciprocated them, and standardized them to represent the level of compliance. The final score ranges from **−**2.60 to 0.74, with a higher score indicating greater compliance.

#### Modelling process

We constructed predictive models using machine-learning techniques to assess the levels of fundamental social motives among Weibo users.

***Labelled data***: In total,184 participants were recruited using an online platform (https://www.wjx.cn/). We collected their scores on the subscales for disease avoidance, mate retention, kin care (family), and kin care (child) motives from the Fundamental Social Motive Scale along with relevant descriptive text as labelled data. The measures, data collection procedures, and characteristics of the sample are presented in the Supplementary Materials.

***Modeling***: We adopted 13 regression algorithms using the *sklearn* package in *Python* 3.10. The modelling process comprised three steps. Firstly, we randomly divided the labelled data into two independent datasets, allocating 80% to the training set and 20% to the testing set. Using the training data, we constructed predictive models in which the algorithms iteratively adjusted internal parameters to capture the relationship between textual features and the corresponding scale scores, minimizing prediction error. The trained models were subsequently applied to the testing set to generate out-of-sample predictions. Secondly, we selected the three top-performing models based on model fit and Pearson correlation coefficients between their predictive outcomes and the true scale scores of the testing set (see Figure S3). Thirdly, we calculated the mean-square error (MSE) of the three models using a five-fold cross-validation method and preserved the model with the smallest MSE, which was the random forest regression model (see Figure S4). Finally, the Weibo text data were imported into the predictive model to obtain the disease avoidance, mate retention, and kin care motives of Weibo users.

#### Materials

***Socioeconomic development***: The HDI scores for each province were obtained from the Subnational Human Development Database (Smits & Permanyer, 2019).

***Control variables***: Besides the control variables in Study 1, cultural tightness has also shown an influence on compliance with epidemic prevention norms (Gelfand et al., 2021). Given that socioeconomic development in China is closely associated with high levels of urbanization and population density (Mao et al., 2016), we did not include population density in the main analytical models (robustness analyses involving population density are reported in Section 2.6 in the Supplementary Materials). Therefore, we controlled for cultural tightness, COVID-19 severity, government response, gender ratio, and proportion of the elderly population at the provincial level. Details of the measures and sources of these covariates are presented in Table S6.

#### Statistical analyses

All statistical analyses were performed using *R* (R Core Team, 2024; Version 4.2). Firstly, we computed the Pearson correlations across the variables. Given the nested data structure, we used the cross-level mediation approach outlined by Pituch and Stapleton (2012). This approach allows for differentiating between individual-level and cluster-level indirect effects in 2-1-1 mediation models and examining them. Building on the theoretical framework presented earlier, we propose that individual-level social motives, rather than their relative position within the cluster, influence the relationship between socioeconomic development and compliance. Therefore, we primarily focus on individual-level indirect effects, with the relevant findings reported in the results section. Cluster-level indirect effects are detailed in the Supplementary Materials. The models were constructed using the *lmer* function from the *lmeTest* package and the *mediate* function from the *mediation* package. We utilized a Monte Carlo simulation with the recommended 20,000 random repetitions to compute 95% confidence intervals (CIs) for indirect effects (Preacher & Selig, 2012). Our data are available on the OSF (https://osf.io/cxha6/?view_only=85d67626c08d414ca73ba87764ef62c2).

### 3.2. Results

The descriptive statistics and the correlation matrix of all variables in Study 2 are presented in Table S7 and Figure S5. As predicted, province-level HDI was negatively related to compliance (*r* = −0.53, *p* = 0.002), disease avoidance (*r* = −0.38, *p* = 0.036), and kin care motives (*r* = −0.57, *p* < 0.001), but not to mate retention motives (*r* = 0.25, *p* = 0.183). Moreover, disease avoidance (*r* = 0.05, *p* < 0.001), mate retention (*r* = 0.03, *p* < 0.001), and kin care motives (*r* = 0.02, *p* = 0.007) were positively correlated with compliance at the individual level.

The ICC of compliance in this study was small (ICC = 0.002). Following Peugh’s (2010) recommendation, we calculated the design effect to be 2.319, exceeding the suggested cut-off point of 2. Under these conditions, it is unreasonable to assume that individuals are independent of each other, as this assumption increases the risk of Type I errors and may lead to biased parameter estimates. Thus, multilevel models were used in the analyses in this study.

**Figure 3.**
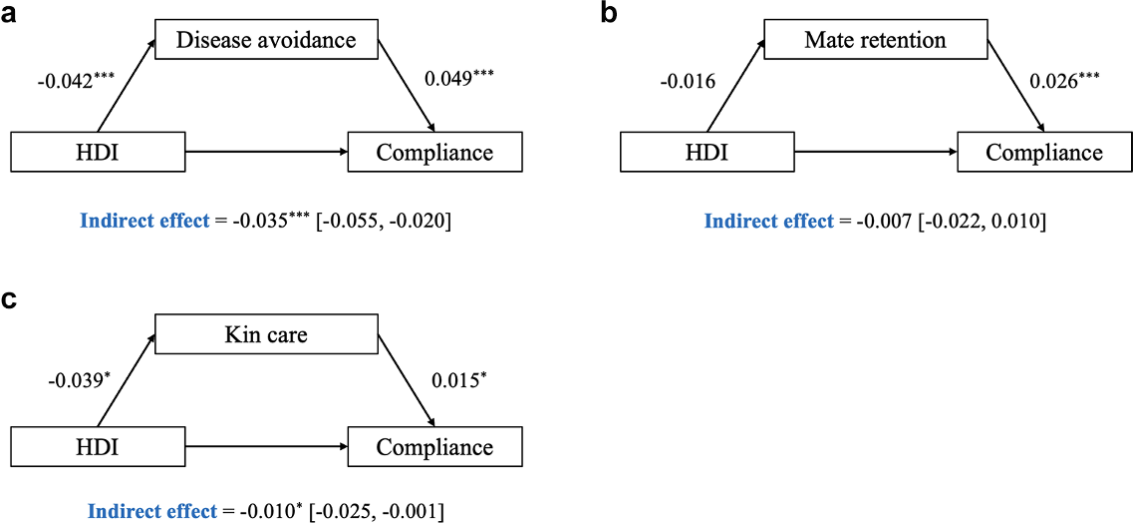
Mediation models in Study 2. (a) Disease avoidance motive, (b) mate retention motive, and (c) kin care motives.

Figure 3 and Table 2 present the results of the HLM analyses. Model 1 included only the control variables. Based on this, Model 2 added the HDI to the intercept term. The HDI was negatively correlated with individual-level disease avoidance (*β* = −0.042, *p* < 0.001) and kin care motives (*β* = −0.039, *p* = 0.045) but not with mate retention motives (*β* = −0.016, *p* = 0.308). Additionally, the HDI was marginally significantly correlated with compliance (*β* = −0.032, *p* = 0.076). The three motives at the individual level were positively correlated with compliance (disease avoidance: *β* = 0.049, *p* < 0.001; mate retention: *β* = 0.026, *p* < 0.001; and kin care: *β* = 0.015, *p* = 0.021), but only kin care motives at the cluster level was positively correlated with compliance (*β* = 0.026, *p* = 0.040). We calculated 95% Monte Carlo CIs, and the results indicated that disease avoidance (indirect effect = −0.035, 95% BootCI [−0.055, −0.020]) and kin care motives (indirect effect = −0.010, 95% BootCI [−0.025, −0.001]) mediated the effect of province-level HDI on compliance, supporting our hypotheses. However, mate retention motives (indirect effect = −0.007, 95% BootCI [−0.022, 0.010]) did not have a significant mediating effect. The results of cluster-level effects are presented in Table S8.

**Table 2.** Hierarchical linear model and multilevel mediation analysis in Study 2 Table 2 long description.

	Compliance		Disease avoidance		Mate retention		Kin care	
	Model 1: CV → C	Model 2: CV + HDI → C	HDI → DA (individual)	DA → C	HDI → MR (individual)	MR → C	HDI → KC (individual)	KC → C
Variables	*β* (*SE*)	*β* (*SE*)	*β* (*SE*)	*β* (*SE*)	*β* (*SE*)	*β* (*SE*)	*β* (*SE*)	*β* (*SE*)
**Level 1 (*N* = 22,588)**								
DA (individual)				0.049***				
				(0.007)				
DA (cluster)				0.014				
				(0.019)				
MR (individual)						0.026***		
						(0.007)		
MR (cluster)						0.015		
						(0.010)		
KC (individual)								0.015*
								(0.007)
KC (cluster)								0.026*
								(0.012)
**Level 2 (*n* = 31)**								
Cultural tightness	−0.038*	−0.023	−0.017	−0.019	−0.021	−0.018	−0.004	−0.023
	(0.014)	(0.015)	(0.012)	(0.015)	(0.015)	(0.014)	(0.016)	(0.013)
Confirmed cases/million	−0.011	−0.011	−0.028**	−0.005	−0.000	−0.011	0.002	−0.013
	(0.015)	(0.013)	(0.011)	(0.015)	(0.013)	(0.012)	(0.014)	(0.012)
CFR	−0.001	0.002	−0.019	0.006	−0.011	0.004	−0.005	0.003
	(0.015)	(0.014)	(0.012)	(0.014)	(0.014)	(0.013)	(0.014)	(0.013)
Government response	0.016	0.016	0.001	0.016	0.018	0.011	0.007	0.014
	(0.014)	(0.013)	(0.010)	(0.012)	(0.013)	(0.012)	(0.013)	(0.011)
Gender ratio	0.040	0.051*	0.050***	0.038	−0.001	0.050✝	0.013	0.045✝
	(0.024)	(0.021)	(0.014)	(0.025)	(0.021)	(0.019)	(0.023)	(0.018)
% Elderly	0.013	0.030	0.047***	0.019	−0.010	0.035	0.007	0.030
	(0.023)	(0.020)	(0.013)	(0.023)	(0.020)	(0.018)	(0.022)	(0.017)
HDI		−0.032✝	−0.042***	−0.023	−0.016	−0.027	−0.039*	−0.013
		(0.015)	(0.010)	(0.017)	(0.015)	(0.013)	(0.016)	(0.015)
*R*^2^_marginal_	0.001	0.002	0.004	0.005	0.001	0.003	0.002	0.003
*R*^2^_conditional_	0.003	0.003	0.004	0.005	0.002	0.003	0.003	0.003
*χ*^2^	8.544**		–	–	–	–	–	–
Indirect effect	–	–	−0.035*** [−0.055, −0.020]		−0.007 [−0.022, 0.010]		−0.010* [−0.025, −0.001]	
Direct effect	–	–	−0.393 [−0.969, 0.180]		−0.470* [−0.915, −0.030]		−0.227 [−0.728, 0.280]	
% Indirect effect			8.152%		–		4.300%	

*Notes*: Gender ratio = (male/female) × 100. % Elderly = proportion of the population aged 65 and above; *β* = standardized coefficient; CV = control variables; C = compliance; *R*^2^_marginal_ = variance explained by fixed factors; *R*^2^_conditional_ = variance explained by both fixed and random factors. ✝*p* < 0.1, *p < 0.05, **p < 0.01, ***p < 0.001. All continuous variables were grand-mean-centred.

The results of the HLMs, including the three sub-dimensions of the HDI, were reported in Table S16. Similar to Study 1, substantial multicollinearity was observed, and the findings should therefore be interpreted with caution.

### 3.3. Discussion

Study 2 used social media text data to enhance the ecological validity of the findings, and the results once again showed that the HDI had an important predictive effect on compliance with epidemic prevention norms. The findings also revealed significant mediating effects of individual-level disease avoidance and kin care motives, supporting our hypotheses. Additionally, both individual- and cluster-level mate retention did not show a mediating effect. One possible reason is the complexity of social media expressions related to mate retention, which typically combine temporal, spatial, and contextual elements to form narratives. Additionally, in the Chinese culture, when people express partner-related motives, they often do so in a veiled manner. To address these limitations, we conducted Study 3.

## 4. Study 3

Study 3 used an online survey with standardized scales to directly measure self-reported compliance and fundamental social motives, addressing the ambiguity often found in social media expressions. This study investigated the relationship between socioeconomic development and compliance at the city level, along with the mediating effects of disease avoidance and familial motives.

### 4.1. Pilot study

An exploratory pilot study was conducted to verify the effectiveness of this compliance measurement tool. In total, 1,692 participants were recruited (56.5% female, aged 18–71 years, *M*_age_ = 31.03, *SD*_age_ = 9.5, 11 cities). The study was approved by the institutional review board of the Ethics Committee of the Institute of Psychology, Chinese Academy of Sciences (reference number: H20016).

Drawing on Murray and Schaller’s (2012) study, we assessed participants’ compliance based on their perceived severity of normative transgressions. Each participant evaluated six scenarios (e.g., ‘During the epidemic, a resident does not wear a mask while engaging in activities within the compound’) on a 6-point scale (1 = not at all serious, 6 = extremely serious). The scale had a Cronbach’s *α* value of 0.647 in a pilot study. Although the reliability value was relatively low, the item statistics indicated that removing any item would reduce the overall reliability. Thus, we proceeded with preliminary correlations between compliance and other variables using a six-item scale.

The Chinese short version of the Fundamental Social Motives Scale (Liu et al., 2023) was used to assess disease avoidance and familial motives among participants (e.g., ‘avoid the disease infection’) on a 6-point scale (1 = not at all strong, 6 = extremely strong; Cronbach’s *α* = 0.675) (see Table S10).

Due to the limited number of groups (11 cities) in this study, a multilevel analysis was not feasible. Therefore, only Pearson’s correlation and linear regression analyses at the individual level were conducted. All statistical analyses were performed using *R* (R Core Team,^2024^; Version 4.2).

The descriptive statistics and the correlation matrix of all variables in the pilot study are shown in Table S11 and Figure S6. Compliance was positively correlated with disease avoidance (*r* = 0.20, *p* < 0.001), mate retention (*r* = 0.14, *p* < 0.001), kin care (family) (*r* = 0.16, *p* < 0.001), and kin care (child) motives (*r* = 0.08, *p* = 0.001). After controlling for sex and age, these four motives remained positively associated with compliance (disease avoidance: *β* = 0.200, *p* < 0.001; mate retention: *β* = 0.148, *p* < 0.001; kin care (family): *β* = 0.163, *p* < 0.001; and kin care (child): *β* = 0.109, *p* < 0.001), replicating the results of Study 2 and supporting the effectiveness of the compliance measurement tool. The analyses of other fundamental social motives are presented in Table S12.

### 4.2. Formal study

#### Participants

We recruited participants online via SurveyPlus (https://www.surveyplus.cn/) from December 18, 2021, to February 20, 2022. A total of 6,548 participants completed the survey. We excluded outliers and removed data from cities with sample sizes below 15, following the guidelines of Zhang and Wang (2010) and Maas and Hox (2004). The final sample consisted of 5,958 participants (50.5% female, age range = 18–78, *M*_age_ = 28.77, *SD*_age_ = 8.27) from 31 cities and 10 provinces. All participants participated and completed the questionnaire voluntarily. The study was approved by the institutional review board of the Ethics Committee of the Institute of Psychology, Chinese Academy of Sciences (reference number: H20016).

#### Materials

***Compliance with epidemic prevention norms***: To enhance the effectiveness of the measurement tool, we incorporated two scenarios based on the pilot study: ‘During the epidemic, a passenger evaded the body temperature check while passing the subway security screening’ and ‘During the epidemic, an employee did not wear a mask when taking the elevator.’ The final scale comprised eight items, each rated on a 6-point Likert scale (1 = not serious at all, 6 = extremely serious), with a Cronbach’s *α* value of 0.855.

***Fundamental social motives***: The Chinese short version of the Fundamental Social Motives used in the formal study was consistent with the pilot study (Cronbach’s *α* = 0.758).

***Control variables***: Results from Study 2 indicate that, in the Chinese context, ecological factors such as government response stringency and cultural orientation had relatively limited effects on compliance with epidemic prevention norms. In addition, the confirmed cases (per million) and the CFR were highly correlated (*r* = 0.910, *p* < 0.001). Given that disease lethality may better capture the severity of the external environment, we used CFR as the indicator of epidemic severity. Therefore, we controlled for individual-level age and gender, as well as city-level CFR (CFR data were taken from December 18, 2021) in this study.

#### Statistical analyses

All statistical analyses were run in *R* (R Core Team, 2024; Version 4.2). Consistent with Study 2, we conducted Pearson correlations and cross-level mediation models and utilized a Monte Carlo simulation with the recommended 20,000 random repetitions to compute 95% CIs for the indirect effects (Preacher & Selig, 2012). Our data are available on the OSF (https://osf.io/cxha6/?view_only=85d67626c08d414ca73ba87764ef62c2).

### 4.3. Results

Compliance was positively correlated with disease avoidance (*r* = 0.31, *p* < 0.001), mate retention (*r* = 0.25, *p* < 0.001), kin care (family) (*r* = 0.29, *p* < 0.001), and kin care (child) motives (*r* = 0.16, *p* = 0.001). The HDI was negatively correlated with compliance (*r* = −0.50, *p* = 0.004) and disease avoidance motives (*r* = −0.42, *p* = 0.018), but not with mate retention (*r* = −0.11, *p* = 0.573), kin care (family) (*r* = −0.16, *p* = 0.401), or kin care (child) motives (*r* = 0.03, *p* = 0.881). The descriptive statistics and the correlation matrix of all variables in Study 3 are shown in Table S11 and Figure S6.

**Figure 4.**
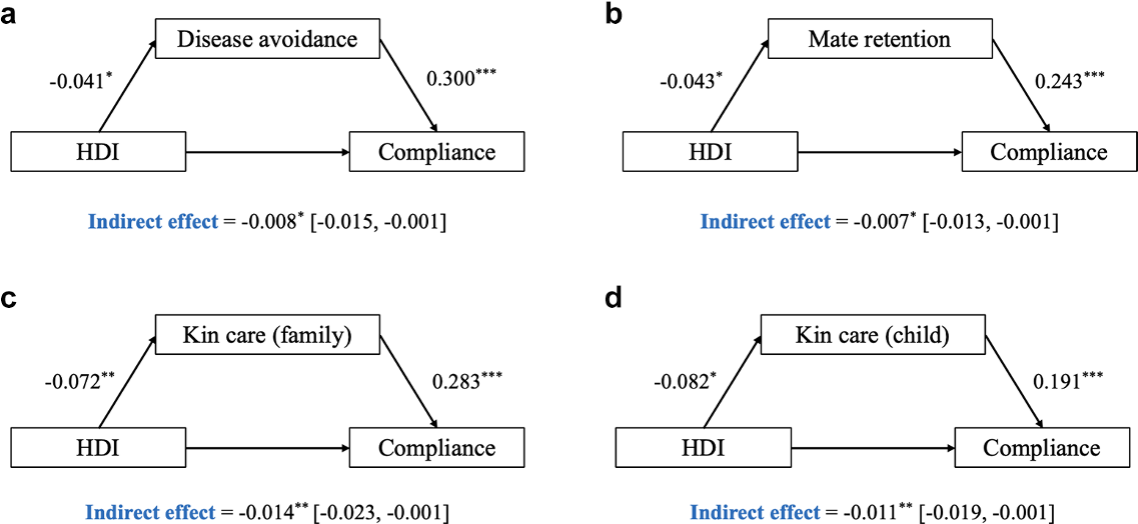
Mediation models in Study 3. (a) Disease avoidance motive, (b) mate retention motive, (c) kin care (family) motive, and (d) kin care (child) motive`.

The ICC of compliance was 0.032, and the corresponding design effect was 6.811 (>2), suggesting that using a multilevel model was sensible (Peugh, 2010). Model 1 included only the control variables. Based on this, Model 2 added the HDI to the intercept term. As shown in Figure 4 and Table 3, a higher HDI was negatively associated with compliance (*p* < 0.001), individual-level disease avoidance (*p* < 0.001), mate retention (*p* < 0.001), kin care (family) (*p* < 0.001), and kin care (child) (*p* < 0.001) motives. All four motive types at the individual level were positively associated with compliance (*p*s < 0.001). We calculated 95% Monte Carlo CIs, and the results indicated that disease avoidance (indirect effect = −0.008, 95% BootCI [−0.015, −0.001]), mate retention (indirect effect = −0.007, 95% BootCI [−0.013, −0.001]), kin care (family) (indirect effect = −0.014, 95% BootCI [−0.023, −0.001]), and kin care (child) motives (indirect effect = −0.011, 95% BootCI [−0.019, −0.001]) mediated the effect of city-level HDI on compliance, supporting our hypotheses. The analyses of cluster-level effects are presented in Table S13.

**Table 3.** Hierarchical linear model and multilevel mediation analysis in Study 3 Table 3 long description.

	Compliance		Disease avoidance		Mate retention		Kin care (family)		Kin care (child)	
	Model 1 CV → C	Model 2 CV + HDI → C	HDI → DA (individual)	DA → C	HDI → MR (individual)	MR → C	HDI → KCF (individual)	KCF → C	HDI → KCC (individual)	KCC → C
Variables	*β* (*SE*)	*β* (*SE*)	*β* (*SE*)	*β* (*SE*)	*β* (*SE*)	*β* (*SE*)	*β* (*SE*)	*β* (*SE*)	*β* (*SE*)	*β* (*SE*)
**Level 1 (*N* = 5,958)**										
Age	−0.009	−0.006	−0.008	−0.004	0.049***	−0.019	0.032*	−0.015	0.357***	−0.074***
	(0.013)	(0.013)	(0.013)	(0.013)	(0.013)	(0.013)	(0.013)	(0.013)	(0.012)	(0.014)
Gender	−0.022✝	−0.022✝	0.011	−0.025*	0.115***	−0.050***	0.049***	−0.036**	0.143***	−0.050***
	(0.013)	−0.022✝	0.011	−0.025*	0.115***	−0.050***	0.049***	−0.036**	0.143***	−0.050***
DA				0.300***						
(individual)				(0.012)						
DA				−0.002						
(cluster)				(0.027)						
MR						0.243***				
(individual)						(0.013)				
MR						0.021				
(cluster)						(0.023)				
KCF								0.283***		
(individual)								(0.012)		
KCF								−0.016		
(cluster)								(0.024)		
KCC										0.191***
(individuals)										(0.014)
KCC (cluster)										−0.004 (0.028)
**Level 2 (*n* = 31)**										
CFR	−0.018	−0.011	0.014	−0.017	−0.023	0.003	−0.035	−0.005	−0.046	−0.003
	(0.054)	(0.036)	(0.018)	(0.036)	(0.022)	(0.031)	(0.028)	(0.036)	(0.039)	(0.035)
HDI		−0.135***	−0.041*	−0.121***	−0.043*	−0.120***	−0.072**	−0.123***	−0.082*	0.119***
		(0.029)	(0.017)	(0.034)	(0.020)	(0.027)	(0.023)	(0.031)	(0.031)	(0.029)
*R*^2^_marginal_	0.001	0.018	0.002	0.107	0.018	0.078	0.009	0.097	0.153	0.050
*R*^2^_conditional_	0.035	0.030	0.004	0.119	0.021	0.087	0.015	0.109	0.169	0.061
*χ*^2^	15.897***		–		–		–		–	
Indirect effect	–	–	−0.008* [−0.015, −0.001]		−0.007* [−0.013, −0.001]		−0.014** [−0.023, −0.001]		−0.011** [−0.019, −0.001]	
Direct effect	–	–	−0.081*** [−0.126, −0.040]		−0.080*** [−0.115, −0.050]		−0.083*** [−0.123, −0.040]		−0.080*** [−0.118, −0.040]	
% Indirect effect			9.360%		8.025%		14.137%		11.641%	

*Notes*: Gender: 0 = female, 1 = male. *β* = standardized coefficient; CV = control variables; C = compliance; HDI = Human Development Index; DA = disease avoidance; MR = mate retention; KCF = kin care (family); KCC = kin care (child); *R*^2^_marginal_ = variance explained by fixed factors; *R^2^*_conditional_ = variance explained by both fixed and random factors. ✝*p* < 0.1, *p < 0.05, **p < 0.01, ***p < 0.001. All continuous variables were grand-mean-centred.

The analyses of other fundamental social motives are also presented in Table S13. The results show that city-level HDI is negatively associated only with individuals’ breakup concern motive and is not significantly related to the other motives. In addition, consistent with previous research (Liu et al., 2023), most motives, except the independence motive, positively predict norm compliance. The breakup concern motive shows a small but significant negative indirect effect (indirect effect = −0.006, 95% CI [−0.010, −0.001]). However, breakup concern is generally a relatively low-salience motive both before and during the pandemic (Pick et al., 2022), suggesting that it is unlikely to represent a primary driver of behaviour in this context. Moreover, the conceptual nature of breakup concern remains debated. Although it is often treated as a sub-dimension of mate retention (Neel et al., 2016), some researchers suggest that it is also closely related to mate-seeking motives and may become activated when relationship stability is uncertain or not yet established (Ko et al., 2019). Therefore, it would be worthwhile to clarify the origins, functional role, and socioecological determinants of breakup concern.

## 5. General discussion

Throughout human history, we have faced a persistent threat from infectious diseases to survival and reproduction (Fabrega, 1997; Fincher et al., 2008). In distinct socioeconomic contexts, people have adopted different strategies to address this threat, such as variations in compliance with epidemic prevention norms. However, little is known about the psychological process underlying these variations. Foundational social motives, evolved for environmental adaptation (Schaller et al., 2017), provide a bridge to understanding the relationship between socioeconomic development environments and compliance behaviour. To better understand these variations and their underlying psychological mechanisms, we conducted three studies. Using the HDI as an indicator of socioeconomic environment, we examined the effects of country-, province-, and city-level HDI on individual compliance and constructed 2-1-1 multilevel mediation models to test the mediating effects of disease avoidance and familial fundamental social motives.

### 5.1. Variations in compliance with epidemic prevention norms over socioeconomic environments

Our three studies consistently found that a lower HDI was associated with greater individual compliance with epidemic prevention norms across methodologically diverse measures. Importantly, the results of Studies 1 and 2 indicated that HDI showed a stronger effect than cultural factors, epidemic severity, and political factors in predicting compliance, suggesting that the socioeconomic development of an environment is one of the key factors underlying regional differences in compliance. This finding aligns with previous studies, indicating that during pandemics, people in environments with lower levels of socioeconomic development exhibit higher levels of compliance with prevention norms, and vice versa (Carlucci et al., 2020; Zhao & Knobel, 2021). The main reason may be attributed to the adaptive responses to environments. The pandemic makes risks and threats of environments with limited resources more severe and salient; to avoid threats and protect their family members as well as themselves, people choose to comply with social norms (Griskevicius et al., 2006; Szekely et al., 2021).

### 5.2. Mediating effects of disease avoidance and familial motives

We found that the lower the socioeconomic level of an environment, the higher the levels of disease avoidance and familial motives. The changes in motivational levels are understandable given the pressure of natural selection on human energy allocation. During pandemics, in environments with limited resources, infections pose a higher risk of mortality, and disease prevention as well as altruistic or cooperative behaviours among blood relatives become more important to individual fitness (Ellis et al., 2009; Fabrega, 1997; Henrich & Muthukrishna, 2021; Murray et al., 2011). People in such environments tend to put more effort into disease avoidance and pursuing familial goals.

Findings of the present study also indicated that both disease avoidance and familial motives positively predicted compliance with prevention norms during pandemics (Studies 2 and 3). These results suggest that under the threat of infectious disease, motivation to avoid infection or care for family members may be more beneficial for survival and reproduction; compliance is an optimal behaviour to achieve these goals. Our findings align with prior research on social norms. Previous studies have established that historical pathogen prevalence, PVD, and concern for relatives’ health contribute to increased normative strictness and compliance during pandemics (Cohen et al., 2020; Murray et al., 2011a; Shook et al., 2020; Stangier et al., 2022). Moreover, fundamental motives, such as disease avoidance and familial motives, have been found to predict compliance with social norms (Liu et al., 2023).

Our main finding was that disease avoidance and familial motives mediated the link between socioeconomic development and compliance with epidemic prevention norms (Studies 2 and 3). Our results provide new insight into why people in different socioeconomic environments show varying levels of compliance during a pandemic. These differences may stem from the varying availability of resources and mortality risks, which shape the cost-benefit analyses of social motives. Specifically, in less developed socioeconomic environments, resource deficiencies exacerbate the death threat posed by infectious diseases, making disease avoidance and cooperation between family members and relatives more valuable in maximizing fitness. These motives guide people to choose more adaptive behaviour – compliance with infectious disease prevention norms during a pandemic in these environments. This finding aligns with research in socioecological psychology (Talhelm & Oishi, 2019), demonstrating that social motives are calibrated to environments to manage social behaviours.

### 5.3. Implications

The present study has several theoretical and practical implications. Firstly, our findings elucidate that regional differences in compliance are behavioural expressions of variation in disease avoidance and familial fundamental social motives, which arise from individuals’ adaptation to different socioeconomic environments during a pandemic. These results offer important insights into the relationship between socioeconomic environments and preventive behaviours against infectious diseases from an evolutionary psychology perspective. Secondly, the present study highlights the role of familial motives in initiating and managing compliance behaviours in the face of infectious disease threats, enriching the theoretical framework of fundamental social motives and social norms. Finally, at a practical level, our findings underscore the necessity of incorporating these motivational factors into the formulation and implementation of epidemic containment policies. Specifically, in regions with high socioeconomic development, emphasizing the importance of long-term family bonds through the media may enhance preventive behaviours against infectious diseases.

### 5.4. Limitations and future directions

Some issues must be noted regarding this study’s limitations and directions for future research. Firstly, this study assumes that resource availability and mortality risk are crucial characteristics of socioeconomic development. However, these two factors have not been explicitly measured. Although the HDI reflects resource availability and mortality risk to some extent, their relationships with the HDI’s three dimensions are complex and difficult to distinguish accurately. For example, life expectancy, a sub-dimension of the HDI, reflects both mortality risk and the availability of health resources. Future research could quantify environmental resource availability and mortality risk and measure individuals’ perceptions of these factors to further refine the mechanism through which the socioeconomic environment shapes compliance behaviour.

In addition, the present study adopted the HDI – an indicator that may be closely linked to survival and reproductive success in environments – to measure levels of socioeconomic development. Life expectancy at birth (health sub-dimension of HDI) and expected years of schooling (education sub-dimension of HDI) reflect conditions for survival, the probability of offspring reaching reproductive age, and opportunities for them to develop competitive abilities. These characteristics of environments represented by the HDI may play a more important role in shaping motives related to maximizing reproductive fitness, such as disease avoidance and familial motives, which guide compliance during epidemics. The socioeconomic environments represented by income/tax-based indices may reflect income disparities and social inequality more. People in such environments are more likely to experience relative deprivation, which increases risk-taking behaviour (Canale et al., 2018). This psychological process may provide an explanation for the findings that mobility in lower-income areas decreased less than in higher-income areas following social distancing declarations (Bonaccorsi et al., 2020; Weill et al., 2020). Future research should investigate whether the effect of the HDI on compliance differs from that of income-based indices during epidemics, while exploring the conditions and motives underlying these differences. This would advance understanding of how socioeconomic environments influence motivational processes under infectious disease threats.

Thirdly, the mediating effects of disease avoidance and familial motives were relatively small (0.007–0.035), suggesting that these motives represent partial mechanisms linking HDI to compliance with epidemic prevention norms rather than fully accounting for the observed associations. Further research would benefit from examining additional potential mechanisms that may contribute to this relationship.

Fourthly, the present study examined the correlations among the HDI, fundamental social motives, and compliance with prevention norms but did not explore causal pathways. It would be worthwhile to collect longitudinal data and utilize more rigorous cross-lagged models to examine the causal mechanisms of this psychological process in the future studies. Additionally, for cross-regional comparisons, formal spatial modelling may offer a valuable extension, providing insights into inter-regional dependencies and helping to further disentangle these effects.

Finally, the present research focuses on individuals’ motivational and behavioural responses under conditions of heightened pathogen threat, examining how these responses vary across different levels of socioeconomic development. An important open question is whether these patterns generalize to non-pandemic contexts, which is critical for strengthening the evolutionary interpretation of our findings. Existing literature has documented that high-development regions tend to have more advanced healthcare systems (Galvan et al., 2020) and more dissolved kin networks (Enke, 2019; Schulz, 2022), which may be associated with lower levels of disease avoidance and familial motives in general contexts. Using non-pandemic data from Pick et al. (2022), we conducted an exploratory analysis that provides preliminary support for this pattern (see Section 4 in the Supplementary Materials for more details). Future research should more systematically examine the relationship between socioeconomic development and social motives under baseline (i.e., non-pandemic) conditions to assess the generalizability of the observed patterns. In addition, disease avoidance and familial motives have been identified as important mechanisms linking socioeconomic development and compliance with epidemic prevention norms. Whether the influence of these motives generalizes to the association between socioeconomic development and domain-general compliance, beyond disease-related norms, remains an important question for future research. Addressing this issue may provide new insights into how individuals trade off different social motives in response to varying task demands under conditions of disease threat.

## Supporting information

10.1017/ehs.2026.10062.sm001 Liu et al. supplementary material Liu et al. supplementary material

## Data Availability

Our data are available on the OSF (https://osf.io/cxha6/?view_only=85d67626c08d414ca73ba87764ef62c2).

## Supplementary material

The supplementary material for this article can be found at https://doi.org/10.1017/ehs.2026.10062.

## Figure 2 Long description

The image consists of four scatter plots comparing the Human Development Index (HDI) with various compliance measures. A) The first plot shows spatial distancing versus HDI. The x-axis is labeled ′HDI′ ranging from 0.5 to 1.0 and the y-axis is labeled ′Spatial Distancing′. A downward trend is observed with a fitted line indicating a negative correlation. B) The second plot displays physical hygiene versus HDI. The x-axis is labeled ′HDI′ ranging from 0.5 to 1.0 and the y-axis is labeled ′Physical Hygiene′. A negative correlation is shown with a fitted line. C) The third plot illustrates policy support versus HDI. The x-axis is labeled ′HDI′ ranging from 0.5 to 1.0 and the y-axis is labeled ′Policy Support′. A downward trend is evident with a fitted line. D) The fourth plot presents comprehensive compliance versus HDI. The x-axis is labeled ′HDI′ ranging from 0.5 to 1.0 and the y-axis is labeled ′Comprehensive Compliance′. The plot shows no significant trend, with the fitted line nearly flat. Each plot includes scattered data points and a shaded area around the fitted line indicating confidence intervals.

Navigate back to Figure 2.

## Table 1 Long description

The table reports standardized coefficients with standard errors from hierarchical linear models run separately in two datasets, with two models per outcome. Outcomes are spatial distancing, physical hygiene, policy support, and compliance; Model 2 adds the country-level human development index compared with Model 1. At the individual level, age and gender are positive and statistically reliable predictors for all four outcomes in both models, and institutional trust is a positive, statistically reliable predictor of compliance. At the country level, individualism shows consistent negative associations, strongest for physical hygiene and policy support, and it remains negative after adding human development index. Government response is positively related to compliance and is statistically reliable in both models, while its links to the other outcomes are weaker and sometimes only marginal. Confirmed cases per million and case fatality rate show mixed, generally small associations, with policy support showing the clearest negative link to confirmed cases in Model 1. Adding human development index yields small improvements in explained variance and modest model fit gains, and human development index itself is negatively associated with each outcome in Model 2. Overall explained variance is modest, and several country-level effects vary by outcome and model, so results should be interpreted as associations rather than causal effects.

Navigate back to Table 1.

## Table 2 Long description

The table reports standardized coefficients with standard errors from multilevel models predicting compliance and testing mediation paths through disease avoidance, mate retention, and kin care, using individual data nested within 31 clusters. In the compliance models, cultural tightness is negatively related to compliance in the controls-only model, but this association is not reliable once human development is added. Human development shows a small negative association with compliance in the model that adds it to controls, and it is strongly negatively related to disease avoidance and modestly negatively related to kin care; its link to mate retention is small and not reliable. At the individual level, disease avoidance and mate retention are each positively associated with compliance, and kin care is also positively associated with compliance with smaller effects; corresponding cluster-level mediator effects are not reliable for disease avoidance and mate retention but are positive for kin care. Among controls, confirmed cases per million, gender ratio, and percent elderly show notable positive or negative associations mainly in the disease avoidance model, while most other control effects are small and inconsistent across outcomes. Model fit indicates very low explained variance overall, with marginal and conditional values close to zero across models. Mediation results indicate a significant indirect pathway from human development through disease avoidance, and a smaller significant indirect pathway through kin care, while the indirect pathway through mate retention is not reliable; direct effects are imprecise and not consistently significant. Interpret results as associations from centered continuous predictors rather than causal effects, and note that significance varies by model and pathway.

Navigate back to Table 2.

## Table 3 Long description

The table reports multilevel models predicting individual compliance and testing whether national development relates to compliance through four individual-level mediators: disease avoidance, mate retention, kin care for family, and kin care for children. In the compliance-only models, national development is linked to lower compliance after accounting for controls, while country case fatality rate shows no clear association. For mediation, higher national development predicts lower disease avoidance, mate retention, and kin care for family, but predicts higher kin care for children. Each mediator at the individual level is positively related to compliance, with the strongest positive link for kin care for children and moderate positive links for disease avoidance, mate retention, and kin care for family. Indirect effects from national development to compliance are statistically significant and negative for all four mediators, indicating that development is associated with lower compliance partly through these pathways. The indirect portion is modest, roughly eight to fourteen percent depending on the mediator, and the remaining direct association between development and compliance stays negative and statistically significant. Age shows little relation to compliance overall, but is positively related to mate retention and kin care for children and negatively related to compliance when kin care for children is included. Gender effects are small but suggest men report slightly lower compliance and higher scores on the mediators, with the direction varying by mediator. Model fit indicates fixed effects explain little variance in the simplest compliance model and more variance in models that include mediators, but results should be interpreted as associations rather than causal effects.

Navigate back to Table 3.

## References

[ref1] Aamir, F. B., Zaidi, S. M. A., Abbas, S., Aamir, S. R., Zaidi, S. N. A., Lal, K. K., & Fatima, S. S. (2022). Non-compliance to social distancing during COVID-19 pandemic: A comparative cross-sectional study between the developed and developing countries. *Journal of Public Health Research*, 11(1), 2614. 10.4081/jphr.2021.2614 PMC8883545 34711046

[ref2] Ackerman, J. M., Hill, S. E., & Murray, D. R. (2018). The behavioral immune system: Current concerns and future directions. *Social and Personality Psychology Compass*, 12(2). 10.1111/spc3.12371

[ref3] Ameye, S. A., Ojo, T. O., Adetunji, T. A., & Awoleye, M. O. (2022). Is there an association between COVID-19 mortality and human development index? The case study of Nigeria and some selected countries. *BMC Research Notes*, 15(1), 186. 10.1186/s13104-022-06070-8 35597995 PMC9123789

[ref4] Arbel, Y., Fialkoff, C., Kerner, A., & Kerner, M. (2022). Do COVID19 infection rates change over time and space? Population density and socio-economic measures as regressors. *Cities*, 120, 103400. 10.1016/j.cities.2021.103400 34334867 PMC8316012

[ref5] Bahrami-rad, D., Beauchamp, J., Henrich, J., & Schulz, J. (2022). Kin-based institutions and economic development. *SSRN Electronic Journal*. 10.2139/ssrn.4200629

[ref6] Bargain, O., & Aminjonov, U. (2020). Trust and compliance to public health policies in times of COVID-19. *Journal of Public Economics*, 192, 104316. 10.1016/j.jpubeco.2020.104316 33162621 PMC7598751

[ref7] Bentler, P. M., & Chou, C.-P. (1987). Practical issues in structural modeling. *Sociological Methods & Research*, 16(1), 78–117. 10.1177/0049124187016001004

[ref8] Bish, A., & Michie, S. (2010). Demographic and attitudinal determinants of protective behaviours during a pandemic: A review. *British Journal of Health Psychology*, 15(4), 797–824. 10.1348/135910710X485826 20109274 PMC7185452

[ref9] Bonaccorsi, G., Pierri, F., Cinelli, M., Flori, A., Galeazzi, A., Porcelli, F., Schmidt, A. L., Valensise, C. M., Scala, A., Quattrociocchi, W., & Pammolli, F. (2020). Economic and social consequences of human mobility restrictions under COVID-19. *Proceedings of the National Academy of Sciences*, 117(27), 15530–15535. 10.1073/pnas.2007658117 PMC7355033 32554604

[ref10] Brandstätter, V., Job, V., & Schulze, B. (2016). Motivational incongruence and well-being at the workplace: Person-job fit, job burnout, and physical symptoms. *Frontiers in Psychology*, 7. 10.3389/fpsyg.2016.01153 27570513 PMC4981689

[ref11] Brown, M., & Sacco, D. F. (2016). Avoiding extraverts: Pathogen concern downregulates preferences for extraverted faces. *Evolutionary Psychological Science*, 2(4), 278–286. 10.1007/s40806-016-0064-6

[ref12] Burnstein, E., Crandall, C., & Kitayama, S. (1994). Some neo-Darwinian decision rules for altruism: Weighing cues for inclusive fitness as a function of the biological importance of the decision. *Journal of Personality and Social Psychology*, 67(5), 773–789. 10.1037/0022-3514.67.5.773

[ref13] Buss, D. M. (1991). Evolutionary personality psychology. *Annual Review of Psychology*, 42, 459–491. 10.1146/annurev.ps.42.020191.002331 2018400

[ref14] Canale, N., Vieno, A., Lenzi, M., Griffiths, M. D., Perkins, D. D., & Santinello, M. (2018). Cross-national differences in risk preference and individual deprivation: A large-scale empirical study. *Personality and Individual Differences*, 126, 52–60. 10.1016/j.paid.2018.01.006

[ref15] Carlucci, L., D’ Ambrosio, I., & Balsamo, M. (2020). Demographic and attitudinal factors of adherence to quarantine guidelines during COVID-19: The Italian model. *Frontiers in Psychology*, 11, 559288. 10.3389/fpsyg.2020.559288 33192820 PMC7609562

[ref16] Chen, C., Frey, C. B., & Presidente, G. (2021). Culture and contagion: Individualism and compliance with COVID-19 policy. *Journal of Economic Behavior & Organization*, 190, 191–200. 10.1016/j.jebo.2021.07.026 34566218 PMC8452375

[ref17] Cialdini, R. B., Reno, R. R., & Kallgren, C. A. (1990). A focus theory of normative conduct: Recycling the concept of norms to reduce littering in public places. *Journal of Personality and Social Psychology*, 58(6), 1015–1026. 10.1037/0022-3514.58.6.1015

[ref18] Cohen, A. K., Hoyt, L. T., & Dull, B. (2020). A descriptive study of COVID-19–related experiences and perspectives of a national sample of college students in spring 2020. *Journal of Adolescent Health*, 67(3), 369–375. 10.1016/j.jadohealth.2020.06.009 PMC7313499 32593564

[ref19] Courtney, E. P., Felig, R. N., & Goldenberg, J. L. (2022). Together we can slow the spread of COVID‐19: The interactive effects of priming collectivism and mortality salience on virus‐related health behaviour intentions. *British Journal of Social Psychology*, 61(1), 410–431. 10.1111/bjso.12487 34312892 PMC8420273

[ref20] Díaz, A., Soriano, J. F., & Beleña, Á. (2016). Perceived vulnerability to disease questionnaire: Factor structure, psychometric properties and gender differences. *Personality and Individual Differences*, 101, 42–49. 10.1016/j.paid.2016.05.036

[ref21] DiGiovanni, C., Conley, J., Chiu, D., & Zaborski, J. (2004). Factors influencing compliance with quarantine in Toronto during the 2003 SARS outbreak. *Biosecurity and Bioterrorism: Biodefense Strategy, Practice, and Science*, 2(4), 265–272. 10.1089/bsp.2004.2.265 15650436

[ref22] Ellickson, R. C. (1999). The evolution of social norms: A perspective from the legal academy. *SSRN Electronic Journal*. 10.2139/ssrn.191392

[ref23] Ellis, B. J., Figueredo, A. J., Brumbach, B. H., & Schlomer, G. L. (2009). Fundamental dimensions of environmental risk: The impact of harsh versus unpredictable environments on the evolution and development of life history strategies. *Human Nature*, 20(2), 204–268. 10.1007/s12110-009-9063-7 25526958

[ref24] Enders, C. K., & Tofighi, D. (2007). Centering predictor variables in cross-sectional multilevel models: A new look at an old issue. *Psychological Methods*, 12(2), 121–138. 10.1037/1082-989X.12.2.121 17563168

[ref25] Enke, B. (2019). Kinship, cooperation, and the evolution of moral systems. *The Quarterly Journal of Economics*, 134(2), 953–1019. 10.1093/qje/qjz001

[ref26] Eom, K., Papadakis, V., Sherman, D. K., & Kim, H. S. (2019). The psychology of proenvironmental support: In search of global solutions for a global problem. *Current Directions in Psychological Science*, 28(5), 490–495. 10.1177/0963721419854099

[ref27] Fabrega, H. (1997). Earliest phases in the evolution of sickness and healing. *Medical Anthropology Quarterly*, 11(1), 26–55. 10.1525/maq.1997.11.1.26 9138767

[ref28] Fincher, C. L., Thornhill, R., Murray, D. R., & Schaller, M. (2008). Pathogen prevalence predicts human cross-cultural variability in individualism/collectivism. *Proceedings of the Royal Society B: Biological Sciences*, 275(1640), 1279–1285. 10.1098/rspb.2008.0094 PMC2602680 18302996

[ref29] Galvan, D., Effting, L., Cremasco, H., & Adam Conte-junior, C. (2020). Can socioeconomic, health, and safety data explain the spread of COVID-19 outbreak on Brazilian Federative units? *International Journal of Environmental Research and Public Health*, 17(23), 8921. 10.3390/ijerph17238921 33266276 PMC7730726

[ref30] Gelfand, M. J., Jackson, J. C., Pan, X., Nau, D., Pieper, D., Denison, E., Dagher, M., Van Lange, P. A. M., Chiu, C.-Y., & Wang, M. (2021). The relationship between cultural tightness–looseness and COVID-19 cases and deaths: A global analysis. *The Lancet Planetary Health*, 5(3), e135–e144. 10.1016/S2542-5196(20)30301-6 33524310 PMC7946418

[ref31] Gigerenzer, G. (2000). *Adaptive thinking: Rationality in the real world* (p. xi,344). Oxford University Press.

[ref32] Griskevicius, V., Ackerman, J. M., Cantú, S. M., Delton, A. W., Robertson, T. E., Simpson, J. A., Thompson, M. E., & Tybur, J. M. (2013). When the economy falters, do people spend or save? Responses to resource scarcity depend on childhood environments. *Psychological Science*, 24(2), 197–205. 10.1177/0956797612451471 23302295

[ref33] Griskevicius, V., Delton, A. W., Robertson, T. E., & Tybur, J. M. (2011). Environmental contingency in life history strategies: The influence of mortality and socioeconomic status on reproductive timing. *Journal of Personality and Social Psychology*, 100(2), 241–254. 10.1037/a0021082 20873933 PMC3556268

[ref34] Griskevicius, V., Goldstein, N. J., Mortensen, C. R., Cialdini, R. B., & Kenrick, D. T. (2006). Going along versus going alone: When fundamental motives facilitate strategic (non)conformity. *Journal of Personality and Social Psychology*, 91(2), 281–294. 10.1037/0022-3514.91.2.281 16881765

[ref35] Gruijters, S. L. K., Tybur, J. M., Ruiter, R. A. C., & Massar, K. (2016). Sex, germs, and health: Pathogen-avoidance motives and health-protective behaviour. *Psychology & Health*, 31(8), 959–975. 10.1080/08870446.2016.1161194 26953783

[ref36] Gul, P., Keesmekers, N., Elmas, P., Köse, F. E., Koskun, T., Wisman, A., & Kupfer, T. R. (2022). Disease avoidance motives trade-off against social motives, especially mate-seeking, to predict social distancing: Evidence from the COVID-19 pandemic. *Social Psychological and Personality Science*, 13(8), 1281–1293. 10.1177/19485506211046462

[ref37] Gurven, M. D. (2018). Broadening horizons: Sample diversity and socioecological theory are essential to the future of psychological science. *Proceedings of the National Academy of Sciences*, 115(45), 11420–11427. 10.1073/pnas.1720433115 PMC6233064 30397108

[ref38] Haselton, M. G., & Nettle, D. (2006). The paranoid optimist: An integrative evolutionary model of cognitive biases. *Personality and Social Psychology Review*, 10(1), 47–66. 10.1207/s15327957pspr1001_3 16430328

[ref39] Hatchett, R. J., Mecher, C. E., & Lipsitch, M. (2007). Public health interventions and epidemic intensity during the 1918 influenza pandemic. *Proceedings of the National Academy of Sciences*, 104(18), 7582–7587. 10.1073/pnas.0610941104 PMC1849867 17416679

[ref40] Henrich, J., & Muthukrishna, M. (2021). The origins and psychology of human cooperation. *Annual Review of Psychology*, 72(1), 207–240. 10.1146/annurev-psych-081920-042106 33006924

[ref41] Hernández Blasi, C. (2023). Whose life do you save? Factors associated with gender differences in altruism toward romantic partners versus genetic relatives. *Psychological Reports*, 126(3), 1430–1444. 10.1177/00332941211050010 35068236

[ref42] Inhorn, M. C., & Brown, P. J. (1990). The anthropology of infectious disease. *Annual Review of Anthropology*, 19, 89–117. 10.1146/annurev.an.19.100190.000513

[ref43] Jarvis, C. I., Van Zandvoort, K., Gimma, A., Prem, K., Auzenbergs, M., O’reilly, K., Medley, G., Emery, J. C., Houben, R. M. G. J., Davies, N., Nightingale, E. S., Flasche, S., Jombart, T., Hellewell, J., Abbott, S., Munday, J. D., Bosse, N. I., Funk, S., & Sun, F., & CMMID COVID-19 working group. (2020). Quantifying the impact of physical distance measures on the transmission of COVID-19 in the UK. *BMC Medicine*, 18(1), 124. 10.1186/s12916-020-01597-8. 32375776 PMC7202922

[ref44] Ji, Y., Ma, Z., Peppelenbosch, M. P., & Pan, Q. (2020). Potential association between COVID-19 mortality and health-care resource availability. *The Lancet Global Health*, 8(4), e480. 10.1016/S2214-109X(20)30068-1 32109372 PMC7128131

[ref45] Jin, Y.-H., Cai, L., Cheng, Z.-S., Cheng, H., Deng, T., Fan, Y.-P., Fang, C., Huang, D., Huang, L.-Q., Huang, Q., Han, Y., Hu, B., Hu, F., Li, B.-H., Li, Y.-R., Liang, K., Lin, L.-K., Luo, L.-S., Ma, J., & Wang, X.-H. (2020). A rapid advice guideline for the diagnosis and treatment of 2019 novel coronavirus (2019-nCoV) infected pneumonia (standard version). *Military Medical Research*, 7(1), 4. 10.1186/s40779-020-0233-6 32029004 PMC7003341

[ref46] Jonason, P. K., Icho, A., & Ireland, K. (2016). Resources, harshness, and unpredictability: The socioeconomic conditions associated with the dark triad traits. *Evolutionary Psychology*, 14(1), 147470491562369. 10.1177/1474704915623699

[ref47] Kaplan, H., Hill, K., Lancaster, J., & Hurtado, A. M. (2000). A theory of human life history evolution: Diet, intelligence, and longevity. *Evolutionary Anthropology: Issues, News, and Reviews*, 9(4), 156–185. 10.1002/1520-6505(2000)9:4<156::AID-EVAN5>3.0.CO;2-7

[ref48] Karakulak, A., Stogianni, M., Alonso‐arbiol, I., Shukla, S., Bender, M., Yeung, V. W. L., Jovanović, V., Musso, P., Scardigno, R., Scott, R. A., Stuart, J., Friehs, M., Toh, Z., Albayrak‐aydemir, N., Arvanitis, A., Buzea, C., Mastrotheodoros, S., Tsang, J., Madeira, F., & Gkomez, A. (2023). The perceived vulnerability to disease scale: Cross‐cultural measurement invariance and associations with fear of COVID‐19 across 16 countries. *Social and Personality Psychology Compass*, 17(11), e12878. 10.1111/spc3.12878

[ref49] Kenrick, D. T., Griskevicius, V., Neuberg, S. L., & Schaller, M. (2010). Renovating the pyramid of needs: Contemporary extensions built upon ancient foundations. *Perspectives on Psychological Science*, 5(3), 292–314. 10.1177/1745691610369469 21874133 PMC3161123

[ref50] Kim, J., & Kwan, M.-P. (2021). The impact of the COVID-19 pandemic on people’s mobility: A longitudinal study of the U.S. from March to September of 2020. *Journal of Transport Geography*, 93, 103039. 10.1016/j.jtrangeo.2021.103039 36569218 PMC9759208

[ref51] Ko, A., Pick, C. M., Kwon, J. Y., Barlev, M., Krems, J. A., Varnum, M. E. W., Neel, R., Peysha, M., Boonyasiriwat, W., Brandstätter, E., Crispim, A. C., Cruz, J. E., David, D., & David, O. A. (2019). Family matters: Rethinking the psychology of human social motivation. *Perspectives on Psychological Science*, 15(1), 173–201. 10.1177/1745691619872986 31791196

[ref52] Kuznetsova, A., Brockhoff, P. B., & Christensen, R. H. B. (2017). lmerTest Package: Tests in linear mixed effects models. *Journal of Statistical Software*, 82(13). 10.18637/jss.v082.i13

[ref53] Lin, T., Harris, E. A., Heemskerk, A., Van Bavel, J. J., & Ebner, N. C. (2021). A multi-national test on self-reported compliance with COVID-19 public health measures: The role of individual age and gender demographics and countries’ developmental status. *Social Science & Medicine*, 286, 114335. 10.1016/j.socscimed.2021.114335 34450390 PMC8378016

[ref54] Liu, R., Zheng, X., Wang, Z., Zhou, M., Weng, J., Li, Y., & Chen, X. (2023). COVID-19 symptoms and compliance: The mediating role of fundamental social motives. *Frontiers in Psychology*, 14, 1093875. 10.3389/fpsyg.2023.1093875 37020914 PMC10067610

[ref55] Maas, C. J. M., & Hox, J. J. (2004). Robustness issues in multilevel regression analysis. *Statistica Neerlandica*, 58(2), 127–137. 10.1046/j.0039-0402.2003.00252.x

[ref56] Makhanova, A., Plant, E. A., & Maner, J. K. (2021). Capturing fluctuations in pathogen avoidance: The situational pathogen avoidance scale. *Evolutionary Psychological Science*, 7(1), 21–38. 10.1007/s40806-020-00256-8 32837865 PMC7424133

[ref57] Mao, Q., Long, Y., & Wu, K. (2016). Spatio-temporal changes of population density and urbanization pattern in China (2000 – 2010). *China City Planning Review*, 25(4).

[ref58] Margraf, J., Brailovskaia, J., & Schneider, S. (2020). Behavioral measures to fight COVID-19: An 8-country study of perceived usefulness, adherence and their predictors. *PLOS ONE*, 15(12), e0243523. 10.1371/journal.pone.0243523 33284865 PMC7721173

[ref59] Martin, J. S., Ringen, E. J., Duda, P., & Jaeggi, A. V. (2020). Harsh environments promote alloparental care across human societies. *Proceedings of the Royal Society B: Biological Sciences*, 287(1933), 20200758. 10.1098/rspb.2020.0758 PMC7482265 32811302

[ref60] Mirahmadizadeh, A., Ghelichi-ghojogh, M., Vali, M., Jokari, K., Ghaem, H., Hemmati, A., Jafari, F., Dehghani, S. S., Hassani, A. H., Jafari, A., & Rezaei, F. (2022). Correlation between human development index and its components with COVID-19 indices: A global level ecologic study. *BMC Public Health*, 22(1), 1549. 10.1186/s12889-022-13698-5 35971079 PMC9376577

[ref61] Mortensen, C. R., Becker, D. V., Ackerman, J. M., Neuberg, S. L., & Kenrick, D. T. (2010). Infection breeds reticence: The effects of disease salience on self-perceptions of personality and behavioral avoidance tendencies. *Psychological Science*, 21(3), 440–447. 10.1177/0956797610361706 20424082

[ref62] Murray, D. R., & Schaller, M. (2012). Threat(s) and conformity deconstructed: Perceived threat of infectious disease and its implications for conformist attitudes and behavior. *European Journal of Social Psychology*, 42(2), 180–188. 10.1002/ejsp.863

[ref63] Murray, D. R., & Schaller, M. (2016). The behavioral immune system: Implications for social cognition, social interaction, and social influence. In J. M. Olson & M. P. Zanna (Eds.), *Advances in experimental social psychology* (Vol. 53, pp. 75–129). Elsevier. 10.1016/bs.aesp.2015.09.002

[ref64] Murray, D. R., Trudeau, R., & Schaller, M. (2011). On the origins of cultural differences in conformity: Four tests of the pathogen prevalence hypothesis. *Personality and Social Psychology Bulletin*, 37(3), 318–329. 10.1177/0146167210394451 21307175

[ref65] Neel, R., Kenrick, D. T., White, A. E., & Neuberg, S. L. (2016). Individual differences in fundamental social motives. *Journal of Personality and Social Psychology*, 110(6), 887–907. 10.1037/pspp0000068 26371400

[ref66] Neuberg, S. L., Kenrick, D. T., & Schaller, M. (2010). Evolutionary social psychology. In *Handbook of social psychology* (5^th^ ed., vol 2, pp. 761–796). John Wiley & Sons, Inc. 10.1002/9780470561119.socpsy002021

[ref67] Pak, A., McBryde, E., & Adegboye, O. A. (2021). Does high public trust amplify compliance with stringent COVID-19 government health guidelines? A multi-country analysis using data from 102,627 individuals. *Risk Management and Healthcare Policy*, 14, 293–302. 10.2147/RMHP.S278774 33542664 PMC7851580

[ref68] Pedersen, M. R. L., Bredahl, T. V. G., Elmose-østerlund, K., & Hansen, A. F. (2022). Motives and barriers related to physical activity within different types of built environments: Implications for health promotion. *International Journal of Environmental Research and Public Health*, 19(15), 9000. 10.3390/ijerph19159000 35897374 PMC9330905

[ref69] Peugh, J. L. (2010). A practical guide to multilevel modeling. *Journal of School Psychology*, 48(1), 85–112. 10.1016/j.jsp.2009.09.002 20006989

[ref70] Pick, C. M., Ko, A., Wormley, A. S., Wiezel, A., Kenrick, D. T., Al-shawaf, L., Barry, O., Bereby-meyer, Y., Boonyasiriwat, W., Brandstätter, E., Crispim, A. C., Cruz, J. E., David, D., David, O. A., Defelipe, R. P., Elmas, P., Espinosa, A., Fernandez, A. M., Fetvadjiev, V. H., & Varnum, M. E. W. (2022). Family still matters: Human social motivation across 42 countries during a global pandemic. *Evolution and Human Behavior*, 43(6), 527–535. 10.1016/j.evolhumbehav.2022.09.003 PMC9534541 36217369

[ref71] Pituch, K. A., & Stapleton, L. M. (2012). Distinguishing between cross- and cluster-level mediation processes in the cluster randomized trial. *Sociological Methods & Research*, 41(4), 630–670. 10.1177/0049124112460380

[ref72] Poirotte, C., & Charpentier, M. J. E. (2020). Unconditional care from close maternal kin in the face of parasites. *Biology Letters*, 16(2), 20190869. 10.1098/rsbl.2019.0869 32097598 PMC7058945

[ref73] Preacher, K. J., & Selig, J. P. (2012). Advantages of Monte Carlo confidence intervals for indirect effects. *Communication Methods and Measures*, 6(2), 77–98. 10.1080/19312458.2012.679848

[ref74] Pyszczynski, T., Lockett, M., Greenberg, J., & Solomon, S. (2021). Terror management theory and the COVID-19 pandemic. *Journal of Humanistic Psychology*, 61(2), 173–189. 10.1177/0022167820959488 38603072 PMC7498956

[ref75] R Core Team. (2024). *R: A language and environment for statistical computing* [Version 4.2.0]. R Foundation for Statistical Computing. https://www.R-project.org/

[ref76] Randall, A. K., Leon, G., Basili, E., Martos, T., Boiger, M., Baldi, M., Hocker, L., Kline, K., Masturzi, A., Aryeetey, R., Bar-kalifa, E., Boon, S. D., Botella, L., Burke, T., Carnelley, K. B., Carr, A., Dash, A., Fitriana, M., Gaines, S. O., & Chiarolanza, C. (2022). Coping with global uncertainty: Perceptions of COVID-19 psychological distress, relationship quality, and dyadic coping for romantic partners across 27 countries. *Journal of Social and Personal Relationships*, 39(1), 3–33. 10.1177/02654075211034236

[ref77] Sacco, D. F., Young, S. G., & Hugenberg, K. (2014). Balancing competing motives: Adaptive trade-offs are necessary to satisfy disease avoidance and interpersonal affiliation goals. *Personality and Social Psychology Bulletin*, 40(12), 1611–1623. 10.1177/0146167214552790 25278107

[ref78] Sawada, N., Auger, E., & Lydon, J. E. (2018). Activation of the behavioral immune system: Putting the brakes on affiliation. *Personality and Social Psychology Bulletin*, 44(2), 224–237. 10.1177/0146167217736046 29020867

[ref79] Schaller, M. (2014). When and how disgust is and is not implicated in the behavioral immune system. *Evolutionary Behavioral Sciences*, 8(4), 251–256. 10.1037/ebs0000019

[ref80] Schaller, M., Kenrick, D. T., Neel, R., & Neuberg, S. L. (2017). Evolution and human motivation: A fundamental motives framework. *Social and Personality Psychology Compass*, 11(6), e12319. 10.1111/spc3.12319

[ref81] Schulz, J. F. (2022). Kin networks and institutional development. *The Economic Journal*, 132(647), 2578–2613. 10.1093/ej/ueac027

[ref82] Sear, R., & Coall, D. (2011). How much does family matter? Cooperative breeding and the demographic transition. *Population and Development Review*, 37, 81–112. 10.1111/j.1728-4457.2011.00379.x 21280366

[ref83] Sear, R., & Mace, R. (2008). Who keeps children alive? A review of the effects of kin on child survival. *Evolution and Human Behavior*, 29(1), 1–18. 10.1016/j.evolhumbehav.2007.10.001

[ref84] Shook, N. J., Sevi, B., Lee, J., Oosterhoff, B., & Fitzgerald, H. N. (2020). Disease avoidance in the time of COVID-19: The behavioral immune system is associated with concern and preventative health behaviors. *PLOS ONE*, 15(8), e0238015. 10.1371/journal.pone.0238015 32817714 PMC7446877

[ref85] Smits, J., & Permanyer, I. (2019). The subnational human development database. *Science Data*, 6, 190038. 10.1038/sdata.2019.38 PMC6413757 30860498

[ref86] Stangier, U., Kananian, S., & Schüller, J. (2022). Perceived vulnerability to disease, knowledge about COVID-19, and changes in preventive behavior during lockdown in a German convenience sample. *Current Psychology*, 41(10), 7362–7370. 10.1007/s12144-021-01456-6 33654348 PMC7906828

[ref87] Stanton, E. A. (2007). The Human Development Index: A History. In *Political Economy Research Institute Working Paper Series* (No. 127; 2007, Vol. *127*, Issue February). https://scholarworks.umass.edu/cgi/viewcontent.cgi?article=1101&context=peri_workingpapers

[ref88] Szekely, A., Lipari, F., Antonioni, A., Paolucci, M., Sánchez, A., Tummolini, L., & Andrighetto, G. (2021). Evidence from a long-term experiment that collective risks change social norms and promote cooperation. *Nature Communications*, 12(1), 5452. 10.1038/s41467-021-25734-w PMC8443614 34526490

[ref89] Talhelm, T., & Oishi, S. (2019). Culture and ecology. In D. Cohen & S. Kitayama (Eds.), *Handbook of cultural psychology* (2^nd^ ed., pp. 119–143). The Guilford Press.

[ref90] Taylor, M., Raphael, B., Barr, M., Agho, K., Stevens, G., & Jorm, L. (2009). Public health measures during an anticipated influenza pandemic: Factors influencing willingness to comply. *Risk Management and Healthcare Policy*, 9. 10.2147/RMHP.S4810 PMC3270909 22312204

[ref91] Tybur, J. M., Lieberman, D., Fan, L., Kupfer, T. R., & De Vries, R. E. (2020). Behavioral immune trade-offs: Interpersonal value relaxes social pathogen avoidance. *Psychological Science*, 31(10), 1211–1221. 10.1177/0956797620960011 32942965 PMC7502680

[ref92] United Nations Development Programme. (2020). *Human development report 2020: The next frontier: Human development and the anthropocene*. United Nations Development Programme. https://hdr.undp.org/content/human-development-report-2020

[ref93] Van Bavel, J. J., Cichocka, A., Capraro, V., Sjåstad, H., Nezlek, J. B., Pavlović, T., Alfano, M., Gelfand, M. J., Azevedo, F., Birtel, M. D., Cislak, A., Lockwood, P. L., Ross, R. M., Abts, K., Agadullina, E., Aruta, J. J. B., Besharati, S. N., Bor, A., Choma, B. L., & Boggio, P. S. (2022). National identity predicts public health support during a global pandemic. *Nature Communications*, 13(1), Article 1. 10.1038/s41467-021-27668-9 PMC8792004 35082277

[ref94] Weill, J. A., Stigler, M., Deschenes, O., & Springborn, M. R. (2020). Social distancing responses to COVID-19 emergency declarations strongly differentiated by income. *Proceedings of the National Academy of Sciences*, 117(33), 19658–19660. 10.1073/pnas.2009412117 PMC7443940 32727905

[ref95] West, S. A., & Gardner, A. (2013). Adaptation and inclusive fitness. *Current Biology*, 23(13), R577–R584. 10.1016/j.cub.2013.05.031 23845249

[ref96] Wolfe, N. D., Dunavan, C. P., & Diamond, J. (2007). Origins of major human infectious diseases. *Nature*, 447(7142), 279–283. 10.1038/nature05775 17507975 PMC7095142

[ref97] Young, S. G., Brown, M., & Sacco, D. F. (2021). Using psychological science to support social distancing: Tradeoffs between affiliation and disease‐avoidance motivations. *Social and Personality Psychology Compass*, 15(5). 10.1111/spc3.12597

[ref98] Zhang, X., & Wang, J. (2010). A study on the sample size problem of hierarchical linear models. *Statistics and Decision*, 2010(15),4–8. 10.13546/j.cnki.tjyjc.2010.15.003

[ref99] Zhao, X., & Knobel, P. (2021). Face mask wearing during the COVID-19 pandemic: Comparing perceptions in China and three European countries. *Translational Behavioral Medicine*, 11(6), 1199–1204. 10.1093/tbm/ibab043 33963866 PMC8240840

