# Supplementary material for: Effects of fundamental social motives on environmental variation of compliance during pandemics
Source: Evol Hum Sci. 2026 Jul 3;8:e31. doi: 10.1017/ehs.2026.10062 (PMC13437989; doi:10.1017/ehs.2026.10062)
Supplement: Supplementary file 1 [file S2513843X26100620sup001.docx]

**Supplementary Materials**

# 1 Study 1

## 1.1 Control variables

**Table S1.** Control variables in Study 1.

| Control variables | Measurements | Sources |
| --- | --- | --- |
| Institutional trust | On a score of 0-10, how much you personally trust each of the institutions below: country’s parliament/government, police, civil service, health system, and government’s effort to handle Coronavirus (0=do not trust at all, 10=complete trust) | COVIDiSTRESS Global Survey (<https://osf.io/z39us/>) |
| COVID-19 severity | Confirmed cases (per million)  Case fatality rate = death cases/confirmed cases  To facilitate model estimation, we applied log transformations to these two variables in the HLMs, specifically log(confirmed cases per million+10) and log(case fatality rate×100+10). | Our World in Data  (<https://ourworldindata.org/>) |
| Government response | Containment and closure (e.g., school closing, workplace closing, stay-at-home requirements), health systems (e.g., public information campaign, contact tracing), and economic response (e.g., income support, fiscal measures) | Oxford COVID-19 Government Response Tracker  (<https://github.com/OxCGRT/covid-policy-tracker>) |
| Individualism | Try to think of those factors that would be important to you in an ideal job; disregard the extent to which they are contained in your present job. How important is it to you to . . . (personal time, freedom, challenge, training, physical conditions, and use of skills) (1=utmost importance to me, 5=very little or no importance). After processing, the final score reflects a country's relative position on the individualistic-collectivist dimension (0=most collectivist; 100=the most individualistic) | Hofstede et al., 2010 |
| Population density | People per sq. km of land area | World bank (https://data.worldbank.org/indicator/EN.POP.DNST?) |

## 1.2 Descriptive statistics and correlations

**Table S2.** Descriptive statistics in Study 1.

| **Data A** | | | **Data B** | | | |  |
| --- | --- | --- | --- | --- | --- | --- | --- |
| **Variables** | *M* | *SD* | **Variables** | *M* | | *SD* | |
| **Level 1 (*N* = 43,244)** | |  | **Level 1 (*N* = 94,657)** | |  | |  |
| Age | 43.76 | 16.20 | Age | 39.89 | | 14.12 | |
| Gender | - | - | Gender | - | | - | |
| Spatial distancing | 8.54 | 1.74 | Institutional trust | 6.05 | | 2.32 | |
| Physical hygiene | 7.91 | 1.89 | Compliance | 5.07 | | 0.87 | |
| Policy support | 7.88 | 2.26 |  |  | |  | |
| **Level 2 (*n* = 53)** | |  | **Level 2 (*n* = 71)** | |  | |  |
| Confirmed cases (per million) | 467.12 | 571.71 | Confirmed cases (per million) | 226.48 | | 258.37 | |
| Case fatality rate | 0.03 | 0.03 | Case fatality rate | 0.03 | | 0.02 | |
| Government response | 63.81 | 8.94 | Government response | 57.70 | | 11.56 | |
| Individualism | 55.03 | 22.50 | Individualism | 57.47 | | 18.09 | |
| Population density (per sq. km) | 245.58 | 866.62 | Population density (per sq. km) | 109.43 | | 184.69 | |
| HDI | 0.87 | 0.09 | HDI | 0.89 | | 0.06 | |
| Health (years) | 79.31 | 4.16 | Health (years) | 79.85 | | 3.33 | |
| Education (years) | 11.29 | 2.14 | Education (years) | 11.83 | | 1.51 | |
| Income ($) | 38061.13 | 18454.75 | Income ($) | 41075.00 | | 14182.06 | |

***Note.*** Gender: 1 = female, 0 = male/other/no reply. Health: life expectancy at birth. Education: mean years of schooling. Income: gross national income (GNI) per capita.

**Figure S1.** Correlation matrices in Study 1. ^*^*p* < 0.05, ^**^*p* < 0.01, ^***^*p* < 0.001. (**A**) Correlation matrix of individual-level variables in Data A (*N* = 43,244). (**B**) Correlation matrix of individual-level variables in DataB (*N* = 94,657). (**C**) Correlation matrix of country-level variables in Data A (*n* = 53). (**D**) Correlation matrix of country-level variables in DataB (*n* = 71).

**Figure S2.** Geographic distribution of compliance across countries in Study 1. (**A**) Geographic distribution of spatial distancing (*n* = 53). (**B**) Geographic distribution of physical hygiene (*n* = 53). (**C**) Geographic distribution of policy support (*n* = 53). (**D**) Geographic distribution of the comprehensive compliance index in Data B (*n* = 71).

## 1.3 Spatial autocorrelation

Moran’s I was computed for each compliance index, providing a direct estimate of spatial dependence, i.e., the degree to which compliance with epidemic prevention norms is correlated across geographic space (Dobson & Gelade, 2012). As shown in Table S3, spatial distancing, physical hygiene, and policy support in Data A, as well as the comprehensive compliance index in Data B, showed positive and significant Moran’s I values (0.153 – 0.373), indicating that geographically proximate nations exhibit similar levels of compliance.

To examine whether the association between the HDI and compliance remains robust after accounting for this spatial interdependence, we included each country’s capital latitude and longitude (Google Developers, n.d.) as covariates. As shown in Table S4, HDI continued to significantly and negatively predict spatial distancing (*β* = -0.120, *p* = 0.015), physical hygiene (*β* = -0.094, *p* = 0.050), and policy support (*β* = -0.156, *p* = 0.023) in Data A, as well as the comprehensive compliance index in Data B (*β* = -0.086, *p* = 0.008), after controlling for geographic location, suggesting that the predictive effect of HDI is robust to spatial dependence.

**Table S3.** Spatial Autocorrelations (Moran’s I) for compliance indices.

| **Dependent variables** | **Moran’s I** | ***p*** |
| --- | --- | --- |
| **Data A (*N* = 43,244, *n* = 53)** |  |  |
| Spatial distancing | 0.210 | < 0.001 |
| Physical hygiene | 0.373 | < 0.001 |
| Policy support | 0.253 | < 0.001 |
| **Data B (*N* = 94,657, *n* = 71)** |  |  |
| Compliance | 0.153 | 0.007 |

**Table S4.** Hierarchical linear models including longitude and latitude in Study 1.

| Variables | Data A (*N* = 43,244, *n* = 53) | | | Data B (*N* = 94,657, *n* = 71) |
| --- | --- | --- | --- | --- |
|  | Spatial distancing | Physical hygiene | Policy support | Compliance |
|  | *β (SE)* | *β (SE)* | *β (SE)* | *β (SE)* |
| Constant | 8.552^***^  (0.048) | 7.934^***^  (0.051) | 7.969^***^  (0.088) | 5.111^***^  (0.029) |
| Level 1 |  |  |  |  |
| Age | 0.114^***^  (0.005) | 0.070^***^  (0.005) | 0.037^***^  (0.005) | 0.141^***^  (0.003) |
| Gender | 0.137^***^  (0.005) | 0.146^***^  (0.005) | 0.090^***^  (0.004) | 0.111^***^  (0.003) |
| Institutional trust |  |  |  | 0.070^***^  (0.004) |
| Level 2 |  |  |  |  |
| Confirmed cases/million | 0.074  (0.051) | 0.007  (0.051) | 0.006  (0.072) | 0.093^*^  (0.041) |
| Case fatality rate | 0.066^*^  (0.028) | 0.035  (0.028) | 0.073^✝^  (0.040) | -0.003  (0.021) |
| Government response | 0.047  (0.030) | 0.032  (0.029) | 0.033  (0.041) | 0.069^*^  (0.032) |
| Individualism | -0.004  (0.035) | -0.133^***^  (0.035) | -0.070  (0.049) | -0.015  (0.028) |
| Population density | 0.023  (0.025) | -0.031  (0.025) | 0.008  (0.035) | -0.000  (0.006) |
| Longitude | -0.073^*^  (0.027) | -0.132^***^  (0.027) | -0.091^*^  (0.038) | -0.089^***^  (0.023) |
| Latitude | -0.112^***^  (0.029) | -0.084^**^  (0.029) | -0.138^**^  (0.041) | -0.073^*^  (0.029) |
| HDI | -0.120^*^  (0.047) | -0.094^*^  (0.047) | -0.156^*^  (0.066) | -0.086^**^  (0.032) |
| *R*^2^_marginal_ | 0.061 | 0.098 | 0.095 | 0.068 |
| *R*^2^_conditional_ | 0.095 | 0.131 | 0.165 | 0.108 |

***Note.*** Gender: 1 = female, 0 = male/other/no reply. *β* = Standardized coefficient. *R^2^_marginal_*: Variance explained by fixed factors. *R^2^_conditional_*: Variance explained by both fixed and random factors. ^✝^*p* < 0.1, ^*^*p* < 0.05, ^**^*p* < 0.01, ^***^*p* < 0.001. All continuous variables were grand-mean-centered.

# 2 Study 2

## 2.1 Lexicons

**Table S5.** Details on lexicons used.

| Lexicons | Dimensions | Num. keywords | Example keywords | Sources |
| --- | --- | --- | --- | --- |
| Noncompliance | Mask wearing | 57 | Don't want to wear a mask | *Guidelines for public protection from COVID-19* (Chinese center for disease control and prevention, 2020)  *COVID-19 Health Education Manual (2nd ed.)* (Chinese health education center, 2020)  Imperial college London YouGov Covid 19 behavior tracker data hub (<https://github.com/YouGov-Data/covid-19-tracker>) |
|  | Hygiene | 32 | No need to wash your hands |  |
|  | Vaccination | 92 | Vaccines don't work |  |
|  | Health monitoring | 99 | Hate to stay at home |  |
|  | Avoid go out | 21 | Go dinner, travel |  |
|  | Social distancing | 79 | Go shopping |  |
| Disease avoidance | Cleaning | 30 | Wash hands, sterilize | The Fundamental Social Motives Inventory (Gao, 2022)  The simplified Chinese version of Linguistic Inquiry and Word Count (Gao et al., 2013)  The Chinese version of Moral Foundations Dictionary (Wu et al., 2019)  the Weibo Five Basic Mood Lexicon (Dong et al., 2015) |
|  | Avoid | 20 | Stay at home |  |
|  | General | 337 | Infectious, epidemic prevention |  |
| Mate retention | Commit | 214 | Closeness, passion of love |  |
|  | Loyal | 103 | Loyal, steadfast |  |
| Kin care | Kin care (family) | 201 | Filial piety, pay back |  |
|  | Kin care (child) | 122 | Raise, bring up |  |

## 2.2 Control variables

**Table S6.** Control variables in Study 2.

| Control variables | Measurements | Sources |
| --- | --- | --- |
| COVID-19 severity | Confirmed cases (per million)  Case fatality rate = death cases/confirmed cases  To facilitate model estimation, we applied log transformations to these two variables in the HLMs, specifically log(confirmed cases per million+10) and log(case fatality rate×100+10). | Sina News  (<https://news.sina.cn/zt_d/yiqing0121>) |
| Government response | Containment and closure (e.g., school closing, workplace closing, stay-at-home requirements), health systems (e.g., public information campaign, contact tracing), and economic response (e.g., income support, fiscal measures) | Oxford COVID-19 Government Response Tracker  (<https://github.com/OxCGRT/covid-policy-tracker>) |
| Cultural tightness | Six items (e.g., “There are many social norms that people are supposed to abide by in this province/city”) developed by Gelfand et al., (2011). After standardizing (z-scores), and adding 3, cultural tightness at the provincial level ranged from 0.85 to 5.00, the higher the score the stronger the level of cultural tightness. | (Chua et al., 2019) |
| Gender ratio | Gender ratio = (male/female)×100 | *Major figures on 2020 population census of China* (National Bureau of Statistics, 2021) |
| % elderly | Proportion of population aged 65 and above |  |
| Population density | People per sq. km of land area |  |

## 2.3 Modelling process

***Labelled data***

*Fundamental social motive**s*. We used the items of disease avoidance, mate retention, kin care (family), and kin care (child) motives from the 45-items Fundamental Social Motives Scale (Gao, 2022). The subscale of disease avoidance contains 4 items (e.g., “I avoid people who might have a contagious illness”), Cronbach’s *α* = 0.700. The subscale of mate retention contains 4 items (e.g., “I do spend much time and energy doing things to keep my partner invested in our relationship”), Cronbach’s *α* = 0.729. The subscale of kin care (family) contains 4 items (e.g., “Caring for family members is important to me”), Cronbach’s *α* = 0.769. The subscale of kin care (child) contains 4 items (e.g., “Taking care of my children is important to me”), Cronbach’s *α* = 0.718. Participants rated the extent to which their current status matched the descriptions on a 6-point Likert scale (1 = strongly disagree, 6 = strongly agree).

*Data collection.* In this study, data were collected between December 21-24, 2022, using online questionnaires (<https://www.wjx.cn/>) and snowball sampling methods. To ensure the quality of the questionnaires, we set up two questionnaires: Questionnaire 1 comprised disease avoidance and mate retention motives, and Questionnaire 2 contained kin care (family) and kin care (child) motives. In each questionnaire, participants were asked to: (1) complete scales of disease avoidance and mate retention motives, or kin care (family) and kin care (child) motives; (2) answer six questions on picture judgments (e.g., judge whether the names of the objects given below correspond to the contents of the pictures), which aimed to reduce the scale's priming effect on motives; (3) describe their thoughts, desires, motives, or related behaviors/actions and experiences about the goal of against diseases, and maintaining a mate relationship (or taking care of one's own relatives and raising one's own children) (if you do not currently have a mate/children, imagine it; no less than 500 words); (4) complete demographic information, including gender, age, marital status, presence of children, and education. Participants in Questionnaire 1 included 92 adults (58.70% female, age range = 19-44, *M_age_* = 23.80, *SD_age_* = 4.80), with 14.13% having children and 16.30% being married. Participants in Questionnaire 2 also included 92 adults (53.26% female, age range = 18-58, *M_age_* = 25.39, *SD_age_* = 6.14), with 33.70% having children and 36.96% being married. The demographics of the participants were similar to the Weibo user profiles in 2020 (54.6% female, *M_age_* = 29.7) (Sina Weibo Data Center, 2021).

***Algorithm fitting***

Figure S3 presents the true motive scores as well as the model-predicted scores of the 20 labeled data, and the Pearson correlation coefficients between the two is shown at the top of the figure. The results suggested that RFR, Bagging and DTR models of disease avoidance, mate retention and kin care motives fitted better to the true values and had relatively high correlation coefficients. We utilized a 5-fold cross-sectional validation to calculate the Mean-Square Error (MSE) of the three models, i.e., the mean of the sum of the squares of the differences between the predicted and true values of all the samples, with a smaller mean square error indicating a better model prediction. Figure S4 shows the order of validation in horizontal coordinates and the MSE of the models from 5-fold cross-validation in vertical coordinates, suggesting that RFR's prediction of disease defense, mate retention, and kin care motives the best effect and the model is optimal.

**Figure S3.** Correlation coefficients of predictive models in Study 2.

**Figure S4.** MSE of 5-fold cross-sectional validation for models in Study 2.

## 2.4 Descriptive statistics and correlations

**Table S7.** Descriptive statistics and correlations in Study 2.

|  | ***M*** | | ***SD*** |
| --- | --- | --- | --- |
| **Level 1 (*N* = 22,588)** | | | |
| Disease avoidance | 0.86 | | 0.04 |
| Mate retention | 0.66 | | 0.04 |
| Kin care | 0.67 | | 0.05 |
| Compliance | - | | - |
| **Level 2 (*n* = 31)** | |  | |
| Cultural tightness | 4.01 | | 0.95 |
| Confirmed cases (per million) | 51.84 | | 208.37 |
| Case fatality rate | 0.01 | | 0.01 |
| Government response | 65.03 | | 5.81 |
| Gender ratio | 106.23 | | 3.94 |
| % elderly | 13.40 | | 2.82 |
| Population density (per sq. km) | 1085.42 | | 1067.75 |
| HDI | 0.80 | | 0.06 |
| Health | 0.92 | | 0.04 |
| Education (years) | 0.69 | | 0.08 |
| Income ($) | 0.81 | | 0.06 |

***Note.*** Gender ratio = (male/female)×100. % elderly = Proportion of the population aged 65 and above. Health: life expectancy at birth. Education: mean years of schooling. Income: gross national income (GNI) per capita.

**Figure S5.** Correlation matrices in Study 2. ^*^*p* < 0.05, ^**^*p* < 0.01, ^***^*p* < 0.001. (**A**) Correlation matrix of individual-level variables (*N* = 22,588). (**B**) Correlation matrix of country-level variables (*n* = 31).

## 2.5 Analyses of cluster-level motives

**Table S****8.** Hierarchical linear model and multilevel mediation analysis of cluster-level motives in Study 2.

|  | | Disease avoidance | | | | Mate retention | | | Kin care | |
| --- | --- | --- | --- | --- | --- | --- | --- | --- | --- | --- |
|  |  | HDI→DA (cluster) | | DA→C | | HDI→MR (cluster) | | MR→C | HDI→KC (cluster) | KC→C |
|  |  | *β* (*SE*) | | *β* (*SE*) | | *β* (*SE*) | | *β* (*SE*) | *β* (*SE*) | *β* (*SE*) |
| Level 1 (*N* = 22,588) | | |  | |  | |  |  |  |  |
| DA (individual) | |  | | 0.049^***^  (0.007) | |  | |  |  |  |
| DA (cluster) | |  | | 0.014  (0.019) | |  | |  |  |  |
| MR (individual) | |  | |  | |  | | 0.026^***^  (0.007) |  |  |
| MR (cluster) | |  | |  | |  | | 0.015  (0.010) |  |  |
| KC (individual) | |  | |  | |  | |  |  | 0.015^*^  (0.007) |
| KC (cluster) | |  | |  | |  | |  |  | 0.026^*^  (0.012) |
| Level 2 (*n* = 31) |  | |  | |  | |  |  |  |  |
| Cultural tightness | | -0.220^*^  (0.006) | | -0.019  (0.015) | | -0.906^***^  (0.021) | | -0.018  (0.014) | -0.271  (0.008) | -0.023  (0.013) |
| Confirmed cases/million | | -0.423^***^  (0.007) | | -0.005  (0.015) | | 0.160^***^  (0.022) | | -0.011  (0.012) | 0.086^*^  (0.008) | -0.013  (0.012) |
| CFR | | -0.171^***^  (0.005) | | 0.006  (0.014) | | -0.514^***^  (0.017) | | 0.004  (0.013) | -0.066^***^  (0.006) | 0.003  (0.013) |
| Government response | | -0.027  (0.006) | | 0.016  (0.012) | | 0.531^***^  (0.019) | | 0.011  (0.012) | 0.052^*^  (0.007) | 0.014  (0.011) |
| Gender ratio | | 0.544^***^  (0.011) | | 0.038  (0.025) | | -0.856^***^  (0.036) | | 0.050^✝^  (0.019) | -0.103^*^  (0.013) | 0.045^✝^  (0.018) |
| % elderly | | 0.310^***^  (0.009) | | 0.019  (0.023) | | -1.364^***^  (0.035) | | 0.035  (0.018) | -0.369^*^  (0.011) | 0.030  (0.017) |
| HDI | | -0.070^**^  (0.008) | | -0.023  (0.017) | | 0.506^***^  (0.028) | | -0.027  (0.013) | -0.155  (0.009) | -0.013  (0.015) |
| *R*^2^_marginal_ | | 0.998 | | 0.005 | | 0.993 | | 0.003 | 0.995 | 0.003 |
| *R*^2^_conditional_ | | 1.000 | | 0.005 | | 1.000 | | 0.003 | 1.000 | 0.003 |
| Indirect effect | | -0.001 [-0.004, 0.000] | | | | 0.007 [-0.002, 0.020] | | | -0.004^*^ [-0.008, -0.001] | |
| Direct effect | | -0.023 [-0.056, 0.010] | | | | -0.027^*^ [-0.054, -0.001] | | | -0.013 [-0.043, 0.020] | |
| % indirect effect | | - | | | | - | | | 23.378% | |

***Note.*** CFR = Case fatality rate. Gender ratio = (male/female)×100. % elderly = Proportion of the population aged 65 and above. *β* = standardized coefficient. ^✝^*p* < 0.1, ^*^*p* < 0.05, ^**^*p* < 0.01, ^***^*p* < 0.001.

## 2.6 Robustness analyses including population density

As shown in Table S9, after controlling for population density, provincial-level HDI remained marginally negatively associated with compliance (with population density: *β* = -0.040, *p* = 0.063; without population density: *β* = -0.032, *p* = 0.076) and significantly negatively associated with both individual-level and cluster-level disease avoidance motive (*β* = -0.039, *p* < 0.001), suggesting that the effect of HDI is robust. However, the association between HDI and individual-level kin care motives was no longer significant (with population density: *β* = -0.030, *p* = 0.145; without population density: *β* = -0.039, *p* = 0.045). This result suggests that the negative association between HDI and kin care motives may substantially overlap with ecological features captured by population density. One possible interpretation, consistent with a life history perspective, is that higher population density intensifies competition for resources, thereby favoring greater investment in competitive ability (i.e., embodied capital) over kinship- and offspring-directed investment (Sng et al., 2017). However, in the Chinese context, provincial-level socioeconomic development is closely associated with population density (*r* = 0.68, *p* < 0.001), making it difficult to disentangle their unique contributions when both variables are included in the model. Future research could address this issue by examining these relationships across different regions or contexts, better isolating the independent effects of HDI and population density on kin care motives.

**Table S9.** Hierarchical linear models including population density in Study 2.

| Variables | Compliance | |  | Disease avoidance | | |
| --- | --- | --- | --- | --- | --- | --- |
|  | Model 1：  CV→C | Model 2：CV+HDI→C | HDI→DA (individual) | | HDI→DA  (cluster) | DA→C |
|  | *β*(*SE*) | *β*(*SE*) | *β*(*SE*) | | *β*(*SE*) | *β*(*SE*) |
| Level 1 (*N* = 22,588) |  |  |  | |  |  |
| DA (individual) |  |  |  | |  | 0.049^***^  (0.007) |
| DA (cluster) |  |  |  | |  | 0.015  (0.019) |
| MR (individual) |  |  |  | |  |  |
| MR (cluster) |  |  |  | |  |  |
| KC (individual) |  |  |  | |  |  |
| KC (cluster) |  |  |  | |  |  |
| Level 2 (*n* = 31) |  |  |  | |  |  |
| Cultural tightness | -0.037^*^  (0.015) | -0.023  (0.015) | -0.017  (0.012) | | -0.048  (0.006) | -0.019  (0.015) |
| Confirmed cases | -0.012  (0.016) | -0.007  (0.014) | -0.030^*^  (0.012) | | -0.381  (0.006) | 0.001  (0.016) |
| Case fatality rate | -0.000  (0.015) | -0.000  (0.014) | -0.017  (0.012) | | -0.180^***^  (0.005) | 0.004  (0.014) |
| Government response | 0.015  (0.015) | 0.013  (0.013) | 0.002  (0.010) | | -0.089  (0.005) | 0.015  (0.012) |
| Gender ratio | 0.042  (0.028) | 0.040  (0.025) | 0.054^**^  (0.019) | | 0.683  (0.010) | 0.025  (0.028) |
| % elderly | 0.014  (0.027) | 0.018  (0.024) | 0.052^**^  (0.019) | | 0.540  (0.009) | 0.005  (0.027) |
| Population density | -0.002  (0.020) | 0.017  (0.019) | -0.005  (0.013) | | 0.120  (0.011) | 0.018  (0.018) |
| HDI |  | -0.040^✝^  (0.018) | -0.039^***^  (0.012) | | -0.557  (0.008) | -0.031  (0.019) |
| *R*^2^_marginal_ | 0.001 | 0.002 | 0.004 | | 0.999 | 0.005 |
| *R*^2^_conditional_ | 0.003 | 0.003 | 0.004 | | 1.000 | 0.005 |
| *χ^2^* | 10.048^**^ | | - | |  | - |

**Table S9. (*continued*)**

| Variables |  | Mate retention | | | |  | | Kin care | | |
| --- | --- | --- | --- | --- | --- | --- | --- | --- | --- | --- |
|  | HDI→MR (individual) | | HDI→MR  (cluster) | MR→C | HDI→KC (individual) | | HDI→KC (cluster) | | KC→C |  |
|  | *β*(*SE*) | | *β*(*SE*) | *β*(*SE*) | *β*(*SE*) | | *β*(*SE*) | | *β*(*SE*) |  |
| Level 1 (*N* = 22,588) |  | |  |  |  | |  | |  |  |
| DA (individual) |  | |  |  |  | |  | |  |  |
| DA (cluster) |  | |  |  |  | |  | |  |  |
| MR (individual) |  | |  | 0.026^***^  (0.007) |  | |  | |  |  |
| MR (cluster) |  | |  | 0.014  (0.010) |  | |  | |  |  |
| KC (individual) |  | |  |  |  | |  | | 0.015^*^  (0.007) |  |
| KC (cluster) |  | |  |  |  | |  | | 0.031^**^  (0.011) |  |
| Level 2 (*n* = 31) |  | |  |  |  | |  | |  |  |
| Cultural tightness | -0.021  (0.015) | | -0.884  (0.021) | -0.017  (0.015) | -0.004  (0.016) | | -0.234^**^  (0.008) | | -0.024^*^  (0.012) |  |
| Confirmed cases | 0.001  (0.015) | | 0.208  (0.023) | -0.007  (0.014) | -0.003  (0.015) | | 0.064^*^  (0.008) | | -0.005  (0.012) |  |
| Case fatality rate | -0.011  (0.014) | | -0.511^***^  (0.017) | 0.002  (0.014) | -0.003  (0.014) | | -0.047^***^  (0.006) | | -0.003  (0.012) |  |
| Government response | 0.018  (0.013) | | 0.483  (0.019) | 0.010  (0.013) | 0.009  (0.014) | | 0.067  (0.007) | | 0.013  (0.010) |  |
| Gender ratio | -0.003  (0.025) | | -0.849  (0.036) | 0.040  (0.023) | 0.024  (0.026) | | -0.071  (0.013) | | 0.021  (0.020) |  |
| % elderly | -0.013  (0.025) | | -1.243  (0.032) | 0.023  (0.023) | 0.019  (0.025) | | -0.349  (0.012) | | 0.007  (0.020) |  |
| Population density | 0.004  (0.019) | | -0.002  (0.038) | 0.016  (0.017) | -0.019  (0.020) | | -0.061^✝^  (0.013) | | 0.027^✝^  (0.014) |  |
| HDI | -0.017  (0.018) | | 0.303^✝^  (0.029) | -0.035^✝^  (0.016) | -0.030  (0.019) | | -0.161  (0.011) | | -0.021  (0.014) |  |
| *R*^2^_marginal_ | 0.001 | | 0.993 | 0.003 | 0.002 | | 0.996 | | 0.003 |  |
| *R*^2^_conditional_ | 0.002 | | 1.000 | 0.004 | 0.003 | | 1.000 | | 0.003 |  |

***Note.*** Gender ratio = (male/female)×100. % elderly = Proportion of the population aged 65 and above. *β* = standardized coefficient. ^✝^*p* < 0.1, ^*^*p* < 0.05, ^**^*p* < 0.01, ^***^*p* < 0.001.

# 3 Study 3

## 3.1 Fundamental social motives scale

**Table S****10.** The Chinese short version of the Fundamental Social Motives Scale.

| **中文** | **English** |
| --- | --- |
| 1. 保护自己不受其他人类的伤害 | Keep myself safe from others |
| 2. 避开疾病传染 | Avoid the disease infection |
| 3. 获得并维护友谊 | Obtain and maintain friendships |
| 4. 成为群体的一部分，对我很重要 | Being part of a group is important for me |
| 5. 获得成功、权力、财富或地位 | Attain success, power, wealth, or status |
| 6. 找伴侣或对象 | Find a romantic or sexual partner |
| 7. 忠于并善待自己的伴侣 | Be faithful and treat my mate well |
| 8. 担心恋人/伴侣离开我 | Worry that my romantic/sexual partner might leave me |
| 9. 担心被排斥 | Worry about being excluded |
| 10. 关爱照顾家人 | Care for my family members |
| 11. 养育照顾子女 | Take care of my children |
| 12. 更愿意一个人待着 | Prefer to spend time alone |

## 3.2 Descriptive statistics and correlations

**Table S11.** Descriptive statistics for Study 3.

| Pilot study | | | | Formal study | | | |
| --- | --- | --- | --- | --- | --- | --- | --- |
| Variables | ***M*** | ***SD*** | **Variables** | | ***M*** | ***SD*** |  |
| Level 1 (*N* = 1,692) |  |  | **Level 1 (*N* = 5,958)** | |  |  |  |
| Age | 31.03 | 9.59 | Age | | 28.77 | 8.27 |  |
| Gender | - | - | Gender | | - | - |  |
| Self-protection | 4.72 | 1.20 | Self-protection | | 4.38 | 1.32 |  |
| Disease avoidance | 5.16 | 0.97 | Disease avoidance | | 5.10 | 1.04 |  |
| Mate retention | 4.98 | 1.11 | Mate retention | | 5.02 | 1.08 |  |
| Backup concern | 3.53 | 1.61 | Backup concern | | 3.71 | 1.61 |  |
| Kin care (family) | 5.17 | 0.84 | Kin care (family) | | 5.17 | 0.91 |  |
| Kin care (child) | 4.21 | 1.66 | Kin care (child) | | 3.96 | 1.73 |  |
| Affiliation (group) | 4.35 | 1.16 | Affiliation (group) | | 4.22 | 1.20 |  |
| Friendship retention | 4.40 | 1.02 | Friendship retention | | 4.51 | 1.07 |  |
| Exclusion concern | 3.60 | 1.40 | Exclusion concern | | 3.65 | 1.37 |  |
| Independent | 3.55 | 1.42 | Independent | | 3.60 | 1.31 |  |
| Status seeking | 3.78 | 1.39 | Status seeking | | 4.02 | 1.33 |  |
| Mate seeking | 2.93 | 1.59 | Mate seeking | | 3.28 | 1.58 |  |
| Compliance | 5.25 | 0.43 | Compliance | | 5.00 | 0.67 |  |
|  |  |  | **Level 2 (*n* = 31)** | |  |  |  |
|  |  |  | Case fatality rate | | 1.06 | 0.08 |  |
|  |  |  | HDI | | 0.81 | 0.05 |  |

***Note.*** Gender: 0 = female, 1 = male.

**Figure S6.** Correlation matrices in Study 3. (**A**) Correlation matrix of individual-level variables in the pilot study (*N* = 1,692). (**B**) Correlation matrix of individual-level variables in the formal study (*N* = 5,958). (C) Correlation matrix of city-level variables in the formal study (*n* = 31). SP = Self-protection, DA = Disease avoidance, MR = Mate retention, BC = Breakup concern, KCF = Kin care (Family), KCC = Kin care (Child), AG = Affiliation (Group), FR = Friendship retention, EC = Exclusion concern, I = Independent, SS = Status seeking, MS = Mate seeking^✝^*p* < 0.1, ^*^*p* < 0.05, ^**^*p* < 0.01, ^***^*p* < 0.001.

## 3.3 Linear regression analyses in the polit study

**Table S12.** Linear regression analyses with compliance as the dependent variable in the polit study.

|  | Self-protection | Disease  avoidance | Mate  retention | Backup  concern | Kin care  (family) | Kin care  (child) | Affiliation  (general) | Friendship  retention | Exclusion  concern | Independent | Status  seeking | Mate  seeking |
| --- | --- | --- | --- | --- | --- | --- | --- | --- | --- | --- | --- | --- |
|  | *β* (*SE*) | *β* (*SE*) | *β* (*SE*) | *β* (*SE*) | *β* (*SE*) | *β* (*SE*) | *β* (*SE*) | *β* (*SE*) | *β* (*SE*) | *β* (*SE*) | *β* (*SE*) | *β* (*SE*) |
| Age | -0.016  (0.024) | -0.013  (0.024) | -0.031  (0.024) | -0.016  (0.024) | -0.026  (0.024) | -0.065^*^  (0.027) | -0.022  (0.024) | -0.028  (0.024) | -0.017  (0.024) | -0.021  (0.025) | -0.020  (0.025) | -0.040  (0.025) |
| Gender | -0.029  (0.024) | -0.025  (0.024) | -0.054^*^  (0.024) | -0.041^✝^  (0.024) | -0.036  (0.024) | -0.041^✝^  (0.024) | -0.039  (0.024) | -0.045^✝^  (0.024) | -0.033  (0.024) | -0.031  (0.024) | -0.032  (0.025) | -0.021  (0.024) |
| Self-protection | 0.061^*^  (0.024) |  |  |  |  |  |  |  |  |  |  |  |
| Disease avoidance |  | 0.200^***^  (0.024) |  |  |  |  |  |  |  |  |  |  |
| Mate retention |  |  | 0.148^***^  (0.024) |  |  |  |  |  |  |  |  |  |
| Backup concern |  |  |  | 0.097^***^  (0.024) |  |  |  |  |  |  |  |  |
| Kin care (family) |  |  |  |  | 0.163^***^  (0.024) |  |  |  |  |  |  |  |
| Kin care (child) |  |  |  |  |  | 0.109^***^  (0.027) |  |  |  |  |  |  |
| Affiliation (general) |  |  |  |  |  |  | 0.098^***^  (0.024) |  |  |  |  |  |
| Friendship retention |  |  |  |  |  |  |  | 0.123^***^  (0.024) |  |  |  |  |
| Exclusion concern |  |  |  |  |  |  |  |  | 0.075^**^  (0.024) |  |  |  |
| Independent |  |  |  |  |  |  |  |  |  | 0.006  (0.025) |  |  |
| Status seeking |  |  |  |  |  |  |  |  |  |  | 0.007  (0.025) |  |
| Mate seeking |  |  |  |  |  |  |  |  |  |  |  | -0.092^***^  (0.025) |
| *F* | 2.95^*^ | 24.31^***^ | 13.19^***^ | 6.13^***^ | 16.35^***^ | 6.49^***^ | 6.38^***^ | 9.48^***^ | 4.10^**^ | 0.90 | 0.91 | 5.41^**^ |
| Adjusted *R^2^* | 0.003 | 0.040 | 0.021 | 0.009 | 0.027 | 0.010 | 0.009 | 0.015 | 0.005 | 0.000 | 0.000 | 0.008 |

***Note.*** Gender: 0 = female, 1 = male. *β* = standardized coefficient.^✝^*p* < 0.1, ^*^*p* < 0.05, ^**^*p* < 0.01, ^***^*p* < 0.001.

## 3.4 Hierarchical linear model and multilevel mediation analyses

**Table S13.** Hierarchical linear model and multilevel mediation analyses in Study 3.

|  | DA | | MR | | | KCF | | | KCC | | |  |
| --- | --- | --- | --- | --- | --- | --- | --- | --- | --- | --- | --- | --- |
|  | HDI→DA (cluster) | DA→C | | HDI→MR (cluster) | MR→C | | HDI→KCF (cluster) | KCF→C | | HDI→KCC (cluster) | KCC→C | |
|  | *β* (*SE*) | *β* (*SE*) | | *β* (*SE*) | *β* (*SE*) | | *β* (*SE*) | *β* (*SE*) | | *β* (*SE*) | *β* (*SE*) | |
| Level 1 (*N* = 5,958) |  |  | |  |  | |  |  | |  |  | |
| Age | -0.000  (0.000) | -0.004  (0.013) | | 0.000 (0.000) | -0.019  (0.013) | | -0.000 (0.000) | -0.015  (0.013) | | 0.000  (0.000) | -0.074^***^  (0.014) | |
| Sex | 0.000  (0.000) | -0.025^*^  (0.013) | | 0.000  (0.000) | -0.050^***^  (0.013) | | -0.000  (0.000) | -0.036^**^  (0.013) | | 0.000  (0.000) | -0.050^***^  (0.013) | |
| DA (individual) |  | 0.300^***^  (0.012) | |  |  | |  |  | |  |  | |
| DA (cluster) |  | -0.002  (0.027) | |  |  | |  |  | |  |  | |
| MR (individual) |  |  | |  | 0.243^***^  (0.013) | |  |  | |  |  | |
| MR (cluster) |  |  | |  | 0.021  (0.023) | |  |  | |  |  | |
| KCF (individual) |  |  | |  |  | |  | 0.283^***^  (0.012) | |  |  | |
| KCF (cluster) |  |  | |  |  | |  | -0.016 (0.024) | |  |  | |
| KCC (individual) |  |  | |  |  | |  |  | |  | 0.191^***^  (0.014) | |
| KCC (cluster) |  |  | |  |  | |  |  | |  | -0.004  (0.028) | |
| Level 2 (*n* = 31) |  |  | |  |  | |  |  | |  |  | |
| CFR | 0.036  (0.021) | -0.017  (0.036) | | -0.355^***^  (0.027) | 0.003  (0.031) | | -0.294  (0.024) | -0.005  (0.036) | | -0.245^***^  (0.021) | -0.003  (0.035) | |
| HDI | -0.662  (0.018) | -0.121^**^  (0.034) | | -0.154^*^  (0.019) | -0.120^***^  (0.027) | | 0.024  (0.019) | -0.123^***^  (0.031) | | 0.034  (0.015) | -0.119^***^  (0.029) | |
| *R*^2^_marginal_ | 0.978 | 0.107 | | 0.925 | 0.078 | | 0.872 | 0.097 | | 0.906 | 0.050 | |
| *R*^2^_conditional_ | 1.000 | 0.119 | | 1.000 | 0.087 | | 1.000 | 0.109 | | 1.000 | 0.061 | |
| Indirect effect | 0.001 [-0.022, 0.020] | | -0.002 [-0.007, 0.000] | | | 0.001 [-0.002, 0.000] | | | -0.000 [-0.002, 0.000] | | |  |
| Direct effect | -0.081^***^ [-0.126. -0.040] | | -0.081^***^ [-0.116, -0.050] | | | -0.083^***^[-0.123, -0.040] | | | -0.079^***^ [-0.117, -0.040] | | |  |

**Table S13. (*continued*)**

|  | BC | | | SP | | | AG | | | FR | | |
| --- | --- | --- | --- | --- | --- | --- | --- | --- | --- | --- | --- | --- |
|  | HDI→BC  (individual) | HDI→BC (cluster) | BC→C | HDI→SP (individual) | HDI→SP (cluster) | SP→C | HDI→AG (individual) | HDI→AG (cluster) | AG→C | HDI→FR (individual) | HDI→FR (cluster) | FR→C |
|  | *β* (*SE*) | *β* (*SE*) | *β* (*SE*) | *β* (*SE*) | *β* (*SE*) | *β* (*SE*) | *β* (*SE*) | *β* (*SE*) | *β* (*SE*) | *β* (*SE*) | *β* (*SE*) | *β* (*SE*) |
| Level 1 (*N* = 5,958) | |  |  |  |  |  |  |  |  |  |  |  |
| Age | -0.057^***^  (0.013) | -0.000 (0.000) | 0.000  (0.013) | -0.021  (0.013) | 0.000  (0.000) | -0.002  (0.013) | 0.018  (0.013) | 0.000  (0.000) | -0.010  (0.013) | 0.033^*^  (0.013) | 0.000  (0.000) | -0.014  (0.013) |
| Sex | 0.139^***^  (0.013) | 0.000 (0.000) | -0.038^**^  (0.013) | -0.029^*^  (0.013) | -0.000  (0.000) | -0.017  (0.013) | 0.017  (0.013) | -0.000  (0.000) | -0.026^*^  (0.013) | 0.075^***^  (0.013) | 0.000  (0.000) | -0.041^**^  (0.013) |
| BC (individual) |  |  | 0.115^***^  (0.013) |  |  |  |  |  |  |  |  |  |
| BC (cluster) |  |  | 0.018 (0.034) |  |  |  |  |  |  |  |  |  |
| SP (individual) |  |  |  |  |  | 0.206^***^  (0.013) |  |  |  |  |  |  |
| SP (cluster) |  |  |  |  |  | -0.024 (0.027) |  |  |  |  |  |  |
| AG (individual) |  |  |  |  |  |  |  |  | 0.209^***^  (0.013) |  |  |  |
| AG (cluster) |  |  |  |  |  |  |  |  | 0.011 (0.017) |  |  |  |
| FR (individual) |  |  |  |  |  |  |  |  |  |  |  | 0.248^***^  (0.012) |
| FR (cluster) |  |  |  |  |  |  |  |  |  |  |  | 0.005 (0.021) |
| Level 2 (*n* = 31) | |  |  |  |  |  |  |  |  |  |  |  |
| CFR | -0.037  (0.029) | -0.144^*^ (0.018) | -0.003  (0.036) | -0.019  (0.032) | -0.185  (0.025) | -0.013  (0.037) | -0.030^*^  (0.013) | -0.466  (0.037) | 0.003  (0.036) | -0.039  (0.031) | -0.305  (0.027) | 0.001  (0.034) |
| HDI | -0.079^**^  (0.024) | -0.514^***^ (0.013) | -0.117^**^  (0.034) | 0.045  (0.026) | 0.442  (0.018) | -0.135^***^  (0.031) | -0.008  (0.013) | 0.170  (0.031) | -0.132^***^  (0.028) | -0.013  (0.026) | 0.224  (0.022) | -0.133^***^  (0.028) |
| *R*^2^_marginal_ | 0.033 | 0.981 | 0.033 | 0.004 | 0.966 | 0.059 | 0.001 | 0.908 | 0.062 | 0.008 | 0.916 | 0.081 |
| *R*^2^_conditional_ | 0.039 | 1.000 | 0.044 | 0.013 | 1.000 | 0.072 | 0.001 | 1.000 | 0.073 | 0.016 | 1.000 | 0.092 |
| Indirect effect (individual) | -0.006^**^ [-0.010, -0.001] | | | 0.006^✝^ [-0.001, 0.010] | | | -0.001 [-0.005, 0.001] | | | -0.002 [-0.011, 0.010] | | |
| Indirect effect (cluster) | -0.006 [-0.029, 0.020] | | | -0.007 [-0.023, 0.010] | | | 0.001 [-0.002, 0.010] | | | 0.001 [-0.006, 0.010] | | |
| Direct effect | -0.078^***^ [-0.123, -0.030] | | | -0.091^***^[-0.131. -0.050] | | | -0.088^***^[-0.126, -0.050] | | | -0.089^***^ [-0.125, -0.050] | | |

**Table S13. (*continued*)**

|  | EC | | | I | | | SS | | | MS | | |
| --- | --- | --- | --- | --- | --- | --- | --- | --- | --- | --- | --- | --- |
|  | HDI→EC (individual) | HDI→EC  (cluster) | EC→C | HDI→I (individual) | HDI→I (cluster) | I→C | HDI→SS (individual) | HDI→SS (cluster) | SS→C | HDI→MS (individual) | HDI→MS (cluster) | MS→C |
|  | *β* (*SE*) | *β* (*SE*) | *β* (*SE*) | *β* (*SE*) | *β* (*SE*) | *β* (*SE*) | *β* (*SE*) | *β* (*SE*) | *β* (*SE*) | *β* (*SE*) | *β* (*SE*) | *β* (*SE*) |
| Level 1 (*N* = 5,958) |  |  |  |  |  |  |  |  |  |  |  |  |
| Age | -0.041^**^  (0.013) | 0.000  (0.000) | -0.002  (0.013) | -0.111^***^  (0.013) | -0.000  (0.000) | -0.007  (0.013) | -0.103^***^  (0.013) | 0.000  (0.000) | -0.000  (0.013) | -0.142^***^  (0.013) | 0.000  (0.000) | 0.005  (0.013) |
| Sex | 0.030^*^  (0.013) | 0.000  (0.000) | -0.026^*^  (0.013) | -0.038^**^  (0.013) | -0.000  (0.000) | -0.022^✝^  (0.013) | 0.116^***^  (0.013) | 0.000  (0.000) | -0.029^*^  (0.013) | 0.213^***^  (0.013) | 0.000  (0.000) | -0.038^**^  (0.013) |
| EC (individual) |  |  | 0.113^***^  (0.013) |  |  |  |  |  |  |  |  |  |
| EC (cluster) |  |  | -0.010 (0.019) |  |  |  |  |  |  |  |  |  |
| I (individual) |  |  |  |  |  | -0.008  (0.013) |  |  |  |  |  |  |
| I (cluster) |  |  |  |  |  | 0.005 (0.024) |  |  |  |  |  |  |
| SS (individual) |  |  |  |  |  |  |  |  | 0.058^***^  (0.013) |  |  |  |
| SS (cluster) |  |  |  |  |  |  |  |  | -0.002  (0.024) |  |  |  |
| MS (individual) |  |  |  |  |  |  |  |  |  |  |  | 0.076^***^  (0.013) |
| MS (cluster) |  |  |  |  |  |  |  |  |  |  |  | -0.044  (0.031) |
| Level 2 (*n* = 31) |  |  |  |  |  |  |  |  |  |  |  |  |
| CFR | -0.011  (0.016) | -0.066  (0.039) | -0.013  (0.037) | -0.022  (0.020) | -0.338  (0.025) | -0.010  (0.037) | -0.020  (0.027) | -0.264  (0.029) | -0.011  (0.037) | -0.056^✝^  (0.029) | -0.457  (0.023) | -0.029  (0.040) |
| HDI | -0.020  (0.015) | -0.539^***^  (0.028) | -0.136^***^  (0.030) | 0.017  (0.018) | -0.389  (0.018) | -0.134^***^  (0.030) | -0.001  (0.023) | -0.288^***^  (0.021) | -0.135^***^  (0.030) | 0.003  (0.024) | -0.043^*^  (0.016) | -0.142^***^  (0.030) |
| *R*^2^_marginal_ | 0.003 | 0.927 | 0.030 | 0.016 | 0.948 | 0.018 | 0.024 | 0.908 | 0.021 | 0.069 | 0.964 | 0.022 |
| *R*^2^_conditional_ | 0.004 | 1.000 | 0.043 | 0.018 | 1.000 | 0.030 | 0.029 | 1.000 | 0.033 | 0.075 | 1.000 | 0.034 |
| Indirect effect (individual) | -0.002 [-0.004, 0.000] | | | -0.000 [-0.000, 0.000] | | | 0.000 [-0.002, 0.000] | | | 0.000 [-0.002, 0.000] | | |
| Indirect effect (cluster) | 0.004 [-0.009, 0.020] | | | -0.001 [-0.013, 0.010] | | | 0.000 [-0.009, 0.010] | | | 0.005 [-0.002, 0.010] | | |
| Direct effect | -0.091^***^ [-0.131, -0.050] | | | -0.090^***^[-0.130, -0.050] | | | -0.090^***^[-0.129, -0.050] | | | -0.095^***^ [-0.134, -0.060] | | |

***Note.*** Gander: 0 = female, 1 = male. *β*: standardized coefficient. SP = Self-protection, DA = Disease avoidance, MR = Mate retention, BC = Breakup concern, KCF = Kin care (family), KCC = Kin care (child), AG = Affiliation (group), FR = Friendship retention, EC = Exclusion concern, I = Independent, SS = Status seeking, MS = Mate seeking, C = Compliance. CFR = Case fatality rate. *R^2^_marginal_*: variance explained by fixed factors; *R^2^_conditional_*: variance explained by both fixed and random factor. ^✝^*p* < 0.1, ^*^*p* < 0.05, ^**^*p* < 0.01, *^***^p* < 0.001.

# 4 Disease avoidance and familial motives and HDI in non-pandemic contexts

Table S14 presents the descriptive statistics of country-level aggregated disease avoidance and familial motives, as well as HDI and its three sub-dimensions, in a non-pandemic context (Pick et al., 2022). As shown in Figure S6, country-level disease avoidance and kin care (family) motives were significantly negatively associated with HDI and its three sub-dimensions (health, education, and income; *r*s > 0.36). This pattern suggests that these associations may generalize beyond pandemic contexts. However, in the non-pandemic context, mate retention and kin care (child) motives were not significantly associated with HDI or its sub-dimensions. The non-significant association between kin care (child) motive and HDI is understandable, as only participants with children responded to the relevant items, resulting in relatively small sample sizes. In several countries, only 10 or fewer participants completed the kin care (child) items, which may limit statistical power (Ko et al., 2019; Pick et al., 2022). In addition, the relationship between socioeconomic development and mate retention remains a matter of debate in general (i.e., non-pandemic) contexts. On the one hand, mate retention has been conceptualized as part of long-term family bonds, which become less tightly maintained as socioeconomic development increases (Enke, 2019; Ko et al., 2019). On the other hand, recent work in cultural evolution has treated romantic love as a distinct phenomenon, showing that it tends to flourish with economic development (Baumard et al., 2022). Reconciling these seemingly contradictory findings and identifying the underlying mechanisms represent an important direction for future research.

**Table S14.** Descriptive statistics for non-pandemic data from Pick et al. (2022).

| Variables | *N* | *M* | *SD* |
| --- | --- | --- | --- |
| Disease avoidance | 32 | 3.98 | 0.42 |
| Mate retention | 32 | 5.68 | 0.30 |
| Kin care (family) | 32 | 5.65 | 0.29 |
| Kin care (child) | 31 | 5.40 | 0.62 |
| HDI | 32 | 0.81 | 0.13 |
| Health (years) | 32 | 77.24 | 5.81 |
| Education (years) | 32 | 10.15 | 2.76 |
| Income ($) | 32 | 29076.56 | 18600.05 |

***Note.*** Health: life expectancy at birth. Education: mean years of schooling. Income: gross national income (GNI) per capita.

**Figure S6.** Correlation matrix of country-level motives, HDI, and its three sub-dimensions in data from Pick et al. (2022).

# 5 Analyses of the three sub-dimensions of the HDI

**Table S15**. Hierarchical linear model results for Study 1 using the three sub-dimensions of HDI.

| **Variables** | **Data A (*N* = 43,244, *n* = 53)** | | | | | | **Data B (*N* = 94,657, *n* = 71)** | |
| --- | --- | --- | --- | --- | --- | --- | --- | --- |
|  | **Spatial distancing** | | **Physical hygiene** | | **Policy support** | | **Compliance** | |
|  | ***β (SE)*** | **VIF** | ***β (SE)*** | **VIF** | ***β (SΕ)*** | **VIF** | ***β (SΕ)*** | **VIF** |
| Constant | 8.578^***^  (0.055) |  | 7.967^***^  (0.067) |  | 8.020^***^  (0.095) |  | 5.110^***^  (0.030) |  |
| **Level 1** |  |  |  |  |  |  |  |  |
| Age | 0.114^***^  (0.005) | 1.01 | 0.070^***^  (0.005) | 1.01 | 0.037^***^  (0.005) | 1.01 | 0.142^***^  (0.003) | 1.01 |
| Gender | 0.137^***^  (0.005) | 1.01 | 0.146^***^  (0.005) | 1.01 | 0.090^***^  (0.004) | 1.01 | 0.111^***^  (0.003) | 1.00 |
| Institutional trust |  |  |  |  |  |  | 0.070^***^  (0.004) | 1.01 |
| **Level 2** |  |  |  |  |  |  |  |  |
| Confirmed cases (million) | 0.079  (0.065) | 4.74 | 0.039  (0.072) | 4.74 | -0.020  (0.085) | 4.73 | 0.079  (0.048) | 3.83 |
| Case fatality rate | 0.015  (0.035) | 1.38 | -0.003  (0.039) | 1.37 | 0.001  (0.046) | 1.36 | -0.024  (0.025) | 1.30 |
| Government response | 0.061^✝^  (0.035) | 1.41 | 0.032  (0.038) | 1.41 | 0.062  (0.046) | 1.40 | 0.099^**^  (0.035) | 1.20 |
| Individualism | 0.031  (0.047) | 2.89 | -0.125^*^  (0.052) | 2.90 | -0.028  (0.062) | 2.92 | -0.006  (0.034) | 2.42 |
| Population density | 0.047  (0.038) | 2.46 | -0.036  (0.042) | 2.46 | 0.022  (0.050) | 2.46 | -0.005  (0.008) | 1.46 |
| Health | 0.030  (0.050) | 3.21 | 0.023  (0.055) | 3.21 | 0.069  (0.065) | 3.21 | -0.042  (0.033) | 2.87 |
| Education | -0.143^*^  (0.054) | 3.60 | -0.130^*^  (0.060) | 3.61 | -0.232^**^  (0.071) | 3.61 | -0.118^**^  (0.036) | 3.45 |
| Income | -0.118  (0.096) | 11.79 | -0.090  (0.106) | 11.80 | -0.102  (0.126) | 11.81 | 0.030  (0.053) | 6.63 |
| *R*^2^_marginal_ | 0.054 |  | 0.091 |  | 0.101 |  | 0.058 |  |
| *R*^2^_conditional_ | 0.099 |  | 0.147 |  | 0.182 |  | 0.107 |  |

***Note.*** Gender: 1 = female, 0 = male/other/no reply. *β* = Standardized coefficient. *R^2^_marginal_*: Variance explained by fixed factors. *R^2^_conditional_*: Variance explained by both fixed and random factors. ^✝^*p* < 0.1, ^*^*p* < 0.05, ^**^*p* < 0.01, ^***^*p* < 0.001. All continuous variables were grand-mean-centered.

**Table S16**. Hierarchical linear model results for Study 2 using the three sub-dimensions of HDI (*N* = 22,588).

| Variables | HDI→C | | HDI→DA (individual) | | HDI→DA (cluster) | | DA→C | |
| --- | --- | --- | --- | --- | --- | --- | --- | --- |
|  | ***β* (*SE*)** | **VIF** | ***β* (*SE*)** | **VIF** | ***β* (*SE*)** | **VIF** | ***β* (*SE*)** | **VIF** |
| Level 1 (*N* = 22, 588) | |  |  |  |  |  |  |  |
| DA (individual) |  |  |  |  |  |  | 0.049^***^  (0.007) | 1.00 |
| DA (cluster) |  |  |  |  |  |  | 0.010  (0.020) | 4.32 |
| MR (individual) |  |  |  |  |  |  |  |  |
| MR (cluster) |  |  |  |  |  |  |  |  |
| KC (individual) |  |  |  |  |  |  |  |  |
| KC (cluster) |  |  |  |  |  |  |  |  |
| Level 2 (*n* = 31) |  |  |  |  |  |  |  |  |
| Cultural tightness | -0.028  (0.018) | 3.74 | -0.022  (0.015) | 4.99 | -0.208  (0.007) | 2.61 | -0.024  (0.018) | 4.08 |
| Confirmed cases | -0.017  (0.015) | 3.03 | -0.035^**^  (0.012) | 3.33 | -0.473  (0.006) | 2.11 | -0.010  (0.018) | 4.33 |
| Case fatality rate | 0.010  (0.016) | 3.64 | -0.008  (0.014) | 4.65 | -0.055  (0.005) | 1.95 | 0.011  (0.016) | 3.65 |
| Government response | 0.015  (0.013) | 2.00 | -0.000  (0.010) | 2.34 | -0.076  (0.005) | 1.56 | 0.016  (0.013) | 2.00 |
| Gender ratio | 0.045  (0.022) | 4.27 | 0.044^**^  (0.015) | 5.13 | 0.528  (0.010) | 3.59 | 0.036  (0.026) | 5.71 |
| % elderly | 0.025  (0.023) | 4.47 | 0.041^*^  (0.016) | 5.71 | 0.476  (0.010) | 4.91 | 0.017  (0.026) | 5.78 |
| Health | 0.003  (0.031) | 10.52 | -0.001  (0.025) | 14.68 | 0.224  (0.012) | 10.16 | 0.004  (0.031) | 10.49 |
| Education | -0.038  (0.026) | 6.16 | -0.044^*^  (0.020) | 9.16 | -0.882  (0.011) | 5.69 | -0.030  (0.029) | 7.12 |
| Income | 0.006  (0.021) | 4.95 | 0.006  (0.018) | 7.03 | 0.219  (0.009) | 3.41 | 0.004  (0.021) | 5.00 |
| *R*^2^_marginal_ | 0.002 |  | 0.005 |  | 0.999 |  | 0.005 |  |
| *R*^2^_conditional_ | 0.003 |  | 0.005 |  | 1.000 |  | 0.005 |  |

**Table S16**. **(*continued*)**

| Variables | HDI→MR (individual) | | | | HDI→MR  (cluster) | | | MR→C | | HDI→KC  (individual) | | HDI→KC (cluster) | | KC→C | |
| --- | --- | --- | --- | --- | --- | --- | --- | --- | --- | --- | --- | --- | --- | --- | --- |
|  | ***β* (*SE*)** | | **VIF** | | ***β* (*SE*)** | | **VIF** | ***β* (*SE*)** | **VIF** | ***β* (*SE*)** | **VIF** | ***β* (*SE*)** | **VIF** | ***β* (*SE*)** | **VIF** |
| Level 1 (N = 22.588) | |  | |  | |  | |  |  |  |  |  |  |  |  |
| DA (individual) |  | |  | |  | |  |  |  |  |  |  |  |  |  |
| DA (cluster) |  | |  | |  | |  |  |  |  |  |  |  |  |  |
| MR (individual) |  | |  | |  | |  | 0.026^***^  (0.007) | 1.00 |  |  |  |  |  |  |
| MR (cluster) |  | |  | |  | |  | 0.020^*^  (0.010) | 2.16 |  |  |  |  |  |  |
| KC (individual) |  | |  | |  | |  |  |  |  |  |  |  | 0.015^*^  (0.007) | 1.00 |
| KC (cluster) |  | |  | |  | |  |  |  |  |  |  |  | 0.024^✝^  (0.012) | 2.02 |
| Level 2 (*n* = 31) |  | |  | |  | |  |  |  |  |  |  |  |  |  |
| Cultural tightness | -0.032^✝^  (0.017) | | 3.97 | | -0.552  (0.025) | | 2.69 | -0.019  (0.016) | 5.71 | -0.008  (0.019) | 3.44 | -0.274^***^  (0.009) | 2.63 | -0.027  (0.016) | 4.21 |
| Confirmed cases | 0.008  (0.014) | | 3.12 | | 0.308^***^  (0.023) | | 2.11 | -0.023^✝^  (0.012) | 3.35 | -0.002  (0.016) | 2.87 | 0.020^*^  (0.008) | 2.11 | -0.018  (0.013) | 3.19 |
| Case fatality rate | -0.021  (0.016) | | 3.89 | | -0.711^***^  (0.019) | | 1.95 | 0.019  (0.015) | 4.89 | 0.001  (0.017) | 3.25 | 0.018^*^  (0.007) | 1.96 | 0.010  (0.015) | 4.09 |
| Government response | 0.024^✝^  (0.012) | | 2.05 | | 0.496  (0.018) | | 1.55 | 0.009  (0.011) | 2.86 | 0.006  (0.014) | 1.91 | 0.051^***^  (0.007) | 1.57 | 0.014  (0.011) | 2.14 |
| Gender ratio | -0.004  (0.020) | | 4.39 | | -0.392  (0.037) | | 3.65 | 0.045^**^  (0.015) | 5.14 | 0.010  (0.026) | 4.13 | -0.161^***^  (0.014) | 3.76 | 0.041  (0.019) | 4.53 |
| % elderly | -0.023  (0.021) | | 4.61 | | -0.923^✝^  (0.036) | | 5.05 | 0.039^*^  (0.016) | 5.96 | 0.004  (0.026) | 4.35 | -0.338^***^  (0.013) | 3.43 | 0.027  (0.019) | 4.79 |
| Health | 0.050^✝^  (0.029) | | 11.21 | | -0.588^*^  (0.044) | | 10.36 | -0.015  (0.027) | 16.93 | -0.003  (0.034) | 9.69 | -0.133^***^  (0.016) | 10.07 | 0.004  (0.028) | 11.94 |
| Education | -0.025  (0.024) | | 6.63 | | 1.112  (0.041) | | 5.71 | -0.033  (0.021) | 9.93 | -0.032  (0.030) | 5.61 | -0.173  (0.015) | 5.67 | -0.024  (0.024) | 7.99 |
| Income | -0.036^✝^  (0.020) | | 5.32 | | -0.545^*^  (0.033) | | 3.49 | 0.024  (0.019) | 7.97 | -0.001  (0.023) | 4.47 | 0.202^**^  (0.012) | 5.09 | 0.010  (0.019) | 5.70 |
| *R*^2^_marginal_ | 0.002 | |  | | 0.994 | |  | 0.003 |  | 0.002 |  | 0.994 |  | 0.003 |  |
| *R*^2^_conditional_ | 0.002 | |  | | 1.000 | |  | 0.003 |  | 0.003 |  | 1.000 |  | 0.003 |  |

***Note.*** DA = Disease avoidance, MR = Mate retention, BC = Breakup concern, KCF = Kin care (family), KCC = Kin care (child). Gender ratio = (male/female)×100. % elderly = Proportion of the population aged 65 and above. *β* = Standardized coefficient. *R^2^_marginal_*: Variance explained by fixed factors. *R^2^_conditional_*: Variance explained by both fixed and random factors. ^✝^*p* < 0.1, ^*^*p* < 0.05, ^**^*p* < 0.01, ^***^*p* < 0.001. All continuous variables were grand-mean-centered.

# References

Baumard, N., Huillery, E., Hyafil, A., & Safra, L. (2022). The cultural evolution of love in literary history. *Nature Human Behaviour*, *6*(4), 506–522. <https://doi.org/10.1038/s41562-022-01292-z>

Chinese centre for disease control and prevention. (2020). *Guidelines for public protection from COVID-19*. People’s Medical Publishing House Co., LTD.

Chinese health education centre. (2020). *COVID-19 Health Education Manual (2nd ed.)*.

Chua, R. Y. J., Huang, K. G., & Jin, M. (2019). Mapping cultural tightness and its links to innovation, urbanization, and happiness across 31 provinces in China. *Proceedings of the National Academy of Sciences*, *116*(14), 6720–6725. <https://doi.org/10.1073/pnas.1815723116>

Dong, Y., Hao, C., Lai, K., Yue, G. (2015). Weibo social moods measurement and validation. *Journal of Psychological Science, 38*(5), 1141-1146. <https://doi.org/10.16719/j.cnki.1671-6981.2015.05.034>

Enke, B. (2019). Kinship, Cooperation, and the Evolution of Moral Systems*. *The Quarterly Journal of Economics*, *134*(2), 953–1019. <https://doi.org/10.1093/qje/qjz001>

Gao, T. (2022). *Effects of childhood environmental stability on fundamental social motives: A moderated mediation model*. [Master’s thesis, University of Chinese Academy of Sciences]. <http://ir.psych.ac.cn/handle/311026/45079>

Gao, R., Hao, B., Li, H., Gao, Y., & Zhu, T. (2013). Developing Simplified Chinese Psychological Linguistic Analysis Dictionary for Microblog. In K. Imamura, S. Usui, T. Shirao, T. Kasamatsu, L. Schwabe, & N. Zhong (Eds.), *Brain and Health Informatics* (Vol. 8211, pp. 359–368). Springer International Publishing. <https://doi.org/10.1007/978-3-319-02753-1_36>

Gelfand, M. J., Raver, J. L., Nishii, L., Leslie, L. M., Lun, J., Lim, B. C., Duan, L., Almaliach, A., Ang, S., Arnadottir, J., Aycan, Z., Boehnke, K., Boski, P., Cabecinhas, R., Chan, D., Chhokar, J., D’Amato, A., Ferrer, M., Fischlmayr, I. C., … Yamaguchi, S. (2011). Differences Between Tight and Loose Cultures: A 33-Nation Study. *Science, 332*(6033), 1100–1104. <https://doi.org/10.1126/science.1197754>

Google Developers. (n.d.). *Country, regional, and world codes*. Google Developers. Retrieved May 20, 2026, from <https://developers.google.com/public-data/docs/canonical/countries_csv>

Hofstede, G. H., Hofstede, G. J., & Minkov, M. (2010). *Cultures and organizations: Software of the mind: intercultural cooperation and its importance for survival (3rd ed)*. McGraw-Hill.

Ko, A., Pick, C. M., Kwon, J. Y., Barlev, M., Krems, J. A., Varnum, M. E. W., Neel, R., Peysha, M., Boonyasiriwat, W., Brandstätter, E., Crispim, A. C., Cruz, J. E., David, D., & David, O. A. (2019). Family Matters: Rethinking the Psychology of Human Social Motivation. Family Matters.

Pick, C. M., Ko, A., Wormley, A. S., Wiezel, A., Kenrick, D. T., Al-Shawaf, L., Barry, O., Bereby-Meyer, Y., Boonyasiriwat, W., Brandstätter, E., Crispim, A. C., Cruz, J. E., David, D., David, O. A., Defelipe, R. P., Elmas, P., Espinosa, A., Fernandez, A. M., Fetvadjiev, V. H., … Varnum, M. E. W. (2022). Family still matters: Human social motivation across 42 countries during a global pandemic. *Evolution and Human Behavior*, *43*(6), 527–535. <https://doi.org/10.1016/j.evolhumbehav.2022.09.003>

National Bureau of Statistics (2021). *Major figures on 2020 population census of China*. China Statistics Press. <https://www.stats.gov.cn/sj/pcsj/rkpc/d7c/202303/P020230301403217959330.pdf>

Sina Weibo Data Center. (2021). *Weibo user development report 2020*. <https://data.weibo.com/report/reportDetail?id=456>

Sng, O., Neuberg, S. L., Varnum, M. E. W., & Kenrick, D. T. (2017). The crowded life is a slow life: Population density and life history strategy. *Journal of Personality and Social Psychology*, *112*(5), 736–754. <https://doi.org/10.1037/pspi0000086>

World bank. (2026). *Population density (people per sq. km of land area)*. https://data.worldbank.org/indicator/EN.POP.DNST?

Wu, S., Yang, C., Zhang, Y. (2019). The Chinese version of moral foundations dictionary: a brief introduction and pilot analysis. *ChinaXiv preprint*. ChinaXiv:201911.00002. <https://doi.org/10.12074/201911.00002>
